# Terahertz linear/non-linear anomalous Hall conductivity of moiré TMD hetero-nanoribbons as topological valleytronics materials

**DOI:** 10.1038/s41598-024-51721-4

**Published:** 2024-01-18

**Authors:** Farzaneh Shayeganfar, Ali Ramazani, Hamidreza Habibiyan, Mohammad Rafiee Diznab

**Affiliations:** 1 Department of Physics and Energy Engineering, Amirkabir University of Technology, Tehran, Iran; 2 Department of Mechanical Engineering, Massachusetts Institute of Technology, Cambridge, MA 02139 USA; 3 Department of Physics and Atmospheric Science, Dalhousie University, Halifax, Nova Scotia, B3H 4R2 Canada

**Keywords:** Applied physics, Condensed-matter physics

## Abstract

Twisted moiré van der Waals heterostructures hold promise to provide a robust quantum simulation platform for strongly correlated materials and realize elusive states of matter such as topological states in the laboratory. We demonstrated that the moiré bands of twisted transition metal dichalcogenide (TMD) hetero-nanoribbons exhibit non-trivial topological order due to the tendency of valence and conduction band states in **K** valleys to form giant band gaps when spin-orbit coupling (SOC) is taken into account. Among the features of twisted WS_2_/MoS_2_ and WSe_2_/MoSe_2_, we found that the heavy fermions associated with the topological flat bands and the presence of strongly correlated states, enhance anomalous Hall conductivity (AHC) away from the magic angle. By band analysis, we showed that the topmost conduction bands from the ± **K**-valleys are perfectly flat and carry a spin/valley Chern number. Moreover, we showed that the non-linear anomalous Hall effect in moiré TMD hetero-nanoribbons can be used to manipulate terahertz (THz) radiation. Our findings establish twisted heterostructures of group-VI TMD nanoribbons as a tunable platform for engineering topological valley quantum phases and THz non-linear Hall conductivity.

## Introduction

The future of quantum technologies hinges on an atomic-level understanding of how stacked and twisted two-dimensional materials interact. Moiré superlattices formed by twisted staking of two-dimensional materials can exhibit various non-trivial quantum phenomena, not intrinsic to the parent materials, which makes them promising candidates for advanced quantum systems^1–4^.

Twisted bilayer graphene (TBG) for instance, has triggered an avalanche of studies following the discovery of the correlated insulating states and superconducting phases at the magic angle^1,5–13^. The fascinating properties of TBG originate from the exceptionally flat moiré bands that are enabled when the twist angle is close to the magic angle. At the magic angle condition, the two Dirac cones are brought sufficiently close to each other that the moiré interlayer potential can force them to hybridize and degenerate into flat bands^13–16^.

**Figure 1:**
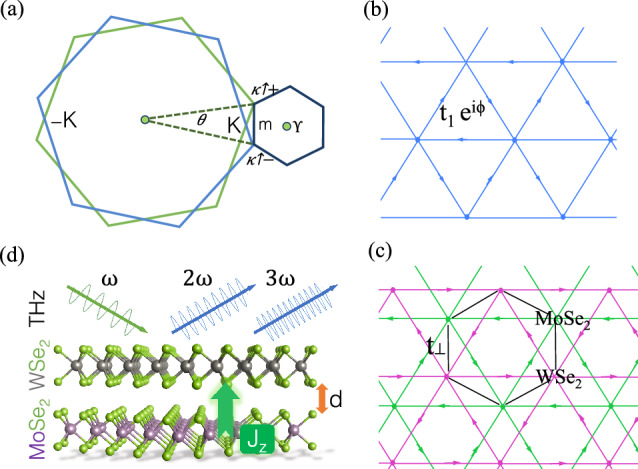
(**a**) The $\kappa ^{\pm }$ points of the moiré Brillouin zone are formed from the **K** points of the monolayer Brillouin zones, which are rotated by $\pm \theta$/2. (**b**) In the absence of an electric field, the triangular lattice sites with nearest neighbour hopping $t_1 = t_1 e^{i\phi }$ are used to model a plain TMD (MX_2_) hetero-nanoribbons with spin-dependent phase $\phi = 2\pi /3 \sigma ^z$. (**c**) In the presence of an electric field, the AB-stacked WSe_2_/MoSe_2_ is modelled by a triangular lattice model similar to (**b**), where a different moiré unit cell center XX’ with opposite spin-orbit coupling phases $\pm 2\pi /3$ are used. The in-plane or intralayer spin-orbit coupling (Eq. 6) and interlayer hopping (Eq. 7) combined together are used to construct an ideal realization of the Kane-Mele model. (**d**) Linear and non-linear optical THz conductivity of WSe_2_/MoSe_2_.

A great deal of attention has also been paid to moiré superlattices of transition metal dichalcogenides (TMDs), formed by Fig. 1 stacking of two different TMD monolayers, MX_2_ and M̃X̃_2_, where M and M̃ are transition metals and X and X̃ are chalcogen atoms ^17–27^.

Moiré TMD superlattices offer a highly tunable platform for studying quantum anomalous Hall phenomena (QAH). Although the moiré bands were believed to be topologically trivial in semiconducting TMD hetero-bilayers^19,28^, recent experimental evidence indicates the non-trivial topology of these bands^29,30^. A well-known example would be the *AB* stacked moiré MoTe_2_/WSe_2_ interface, which displays valley-polarized QAH states^29–32^.

The non-trivial electron wave-function topology can find application in broadband long-wavelength photo-detection and terahertz technologies through the non-linear Hall effect (NHE)^33^. Moiré superlattices, such as TBG^34^ and twisted bilayer WSe_2_^35^ can exhibit NHE, which is characterized by the generation of a second harmonic Hall voltage in response to an injection current in time-reversal symmetric conditions. As such, moiré superlattices seem to have the potential to be used in frequency detection and rectification technologies^33,36^.

In two-dimensional (2D) heterostructures, the coupling of atomic layers cause interlayer electron−electron scattering processes, which are essential for describing their physical properties and complex phases such as correlated insulating ^2^, unconventional superconducting^1,11,37,38^. For twisted bilayer graphene (TBG), van Hove singularities (vHs) in the density of electronic states (DOS) represent the interaction between electrons of different layers ^15,39,40^. More recently, Tao et al.^41^ reported that ultra-flat bands have been created in the large energy barrier moiré superlattice of semiconductors, even for large twist angles by the interlayer interaction due to defect-like states under twisting. Their findings reveal that the appearance of ultra-flat bands in these structures is independent of the twist angle unlike in bilayer graphene, and it can be controlled by external gate fields ^41^.

Here, we propose that moiré superlattices formed by twisted stacking of TMD nanoribbons hold promise for next-level terahertz applications due to their topological properties. Our study is centred around twisted nanoribbons of WS_2_/MoS_2_ and WSe_2_/MoSe_2_. We found that (1) by taking the spin-orbit coupling of moiré TMD hetero-nanoribbons into account, topological band edges and midgap states due to twist angle disorder emerge, and, (2) the linear and non-linear anomalous valley Hall conductivity (AVHC) of twisted TMD hetero-nanoribbons is their unconventional quantum transport response of topological nature, and, (3) the magnitude and sign of the AVHC of these heterostructures is tunable by spin-orbit coupling strength, electric field and twist angle. The calculated Hall responsivity sheds light on the potential of using the intrinsic quantum properties of twisted TMD hetero-nanoribbons as quantum materials for non-linear applications such as THz detection and rectification.

## Computational methods

We consider both armchair (AC) and zigzag (ZZ) structures of WS_2_/MoS_2_ and WSe_2_/MoSe_2_ hetero-nanoribbons at small and large twist angles of ($\theta$ = 0^∘^, 1.1^∘^, 5^∘^, 9^∘^, 15^∘^ and 21^∘^) as shown in Fig. 1. These particular angles are chosen due to existence of experimental data of twisted bilayer graphene and hetero-bilayers^2^. Moreover, we consider defective and strained TMD hetero-nanoribbons. These were studied under uniaxial Y-arc strain with $\delta = 0.05, 0.2$ displacements.

Recently, Gilardoni et al.^42^ reported that a 2*H*-type bilayer TMD with a transition metal atom bound to six chalcogen atoms, shows the crystallographic point group D_3d_, while monolayer TMD exhibits a D_3h_ symmetry. We constructed the moiré pattern by stacking layered group VI TMDs such as MoS_2_ and WS_2_ in 2*H*-type phase with the crystallographic point group D_3d_.

We implemented a tight-binding (TB) model to compute the electronic properties and optical conductivity of the hetero-nanoribbons of WS_2_/MoS_2_ and WSe_2_/MoSe_2_ as a function of the twist angle. As a first step, we created a twisted TMD hetero-nanoribbon, starting from 3R stacked bilayers. The top layer, in which its atoms are arranged directly above the corresponding chalcogen and metal atoms of the bottom layer, was rotated by an angle $\theta$ around the axis perpendicular to the plane of the bilayer, as shown in Fig. 1. It is worth noting that if we construct finite-size nanoribbons in one direction, passivation of a few dangling bonds on the edge of the system is obligatory. Furthermore, to remove these dangling bonds in our TB model we set up lattice neighbours attribution, by using the minimum lattice neighbours method.

### Tight binding model and electronic structure calculation of TMD nanoribbons

We began by writing TB Hamiltonian for non-interacting homo-bilayers then included the intra- and inter-layer hopping terms. The moiré cell vectors ${\textbf{t}}_{\textbf{1}}$ and ${\textbf{t}}_{\textbf{2}}$ were expressed as^43^:

1 $$\begin{aligned} {\textbf{t}}_{\textbf{1}}\ =\ n{\textbf{a}}_{\textbf{1}}+\ m{\textbf{a}}_{\textbf{2}},\ \ \ {\textbf{t}}_{\textbf{2}}\ =\ -m{\textbf{a}}_{\textbf{1}}+(n+m){\textbf{a}}_{\textbf{2}} \end{aligned}$$

where ${\textbf{a}}_{\textbf{1}}$ and ${\textbf{a}}_{\textbf{2}}$ were primitive lattice vectors with a lattice constant of the monolayer material and *m*, *n* were integers, defined as ^43^:

2 $$\begin{aligned} {\textbf{a}}_{\textbf{1}}=\ \frac{a}{2}\ \left( \sqrt{3},1,0\right) \;\ {\textbf{a}}_{\textbf{2}}=\ \frac{a}{2}\ \left( \sqrt{3},-1,0\right) \end{aligned}$$

and the twist angle and the number of atoms in the cell were:

3 $$\begin{aligned} N_{\textrm{atom}} = 6\,\left( n^2 + nm + m^2 \right) ,\nonumber \\ \cos {\theta }=\ \frac{n^2+4nm+\ m^2}{2(n^2+nm+\ m^2)} \end{aligned}$$

For hetero-bilayers, we constructed two TMD monolayers with the same chalcogen atom species with 1% lattice mismatch, and generated a commensurate moiré cell for the twisted structure. We used the same approach for homo-bilayers. To generate moiré cells for these heterostructures of TMD, the approach of Zeller and G$\ddot{\textrm{u}}$nther^44^ were applied. In their model, the moiré vectors $\acute{{\textbf{t}}_{\textbf{1}}}$ and $\acute{{\textbf{t}}_{\textbf{2}}}$, are defined as:

4 $$\begin{aligned} \acute{{\textbf{t}}_{\textbf{1}}}\ =\ n\acute{{\textbf{a}}_{\textbf{1}}}+\ m\acute{{\textbf{a}}_{\textbf{2}}},\ \ \ \acute{{\textbf{t}}_{\textbf{2}}}\ =\ -m\acute{{\textbf{a}}_{\textbf{1}}}+(n-m)\acute{{\textbf{a}}_{\textbf{2}}} \end{aligned}$$

where $\acute{{\textbf{a}}_{\textbf{1}}}$ and $\acute{{\textbf{a}}_{\textbf{2}}}$ are the primitive lattice vectors of the monolayer. We obtained the equilibrium lattice constants of monolayers, *a*, from DFT calculations of Ref. ^45^. The lattice vectors would take the form of:

5 $$\begin{aligned} \acute{{\textbf{a}}_{\textbf{1}}}=\ a\ \left( 1,1,0\right) \;\ \acute{{\textbf{a}}_{\textbf{2}}}=\ \frac{a}{2}\ \left( -1,\sqrt{3},0\right) \end{aligned}$$

where *m* and *n* were integers determined from the numerical solution of a Diophantine equation^43^.

The effective tight-binding model has been adjusted by considering the electric field and exchange field in the effective Hamiltonian, where it highlights the interplay between topology and interaction effects in TMD hetero-nanoribbons. To acquire a topological transition (as a function of the electric field), we construct the interlayer coupling, which is extracted from a Kane-Mele model following Eqs. (6–8).

Moreover, we figure out that the observed topological transition in TMD hetero-nanoribbons is due to coupling to the states in the second layer, which is induced by pseudo-fields and perpendicular electric fields as discussed by references^46,47^. To our knowledge, there is no simple tight-binding model for explaining this topological transition. Herein, we construct an effective model on Eqs. (6–8), indicating topologically non-trivial bands in a hopping model. We take into account the below steps to give an effective model for moiré TMD hetero-nanoribbons^48^.

**Step 1: Topological transition in TMD hetero-nanoribbons-** In the absence of electric field, triangular lattice sites with nearest neighbour hopping $t_1 = t_1 e^{i\phi }$ is used to describe a model of plain TMD (MX_2_) hetero-nanoribbons with spin-dependent phase $\phi = 2\pi /3 \sigma ^z$ as represented in Fig. 1b. In the presence of an electric field, the AB-stacked WSe_2_/MoSe_2_ is modelled by a similar triangular lattice model as shown in Fig. 1c, but with different moiré unit cell centers XX’ with opposite spin-orbit coupling phases $2\pi /3$. The in-plane or intralayer spin-orbit coupling (Eq. 6) and interlayer hopping (Eq. 7) combined together are used to construct an ideal realization of the Kane-Mele model. The Kane-Mele spin-orbit coupling term indicates the intralayer hopping in the TMD hetero-nanoribbons as^48^:

6 $$\begin{aligned} H = \sum _{<<ij>>l\sigma } t_le^{il\sigma ^z\nu_{<<ij>>}\phi} \ {c^\dagger_{il\sigma }}{c_{jl\sigma },} \end{aligned}$$

where $\sigma ^z = \pm 1$ represents the spin, $l = \pm 1$ is the layer index, and $<<ij>>$ takes into account the next-nearest neighbour on the honeycomb lattice where $\nu_{<<ij>>} = \pm 1$ depends on the direction. In this equation, *c *and *c†* are fermionic annihilation/creation operators. 

**Step 2:** The nearest neighbour hopping on the honeycomb lattice is responsible for the interlayer hopping, where the C3 symmetry constrains the possible complex phases of the interlayer hopping^46^. For the tight-binding model, the interlayer hopping yields^48^:

7 $$\begin{aligned} H_\perp = t_\perp \sum _{<ij>\sigma }e^{i\frac{2\pi}{3}\nu _{<ij>}}{c^\dagger_{il\sigma }}{c_{jl\sigma }} \end{aligned}$$

**Step 3:** By applying a perpendicular electric field (V), the states of WSe_2_ layer shift upward as^48^:

8 $$\begin{aligned} H_V = (V+\Delta )\sum _{i}n_{i,l=2}. \end{aligned}$$

Here $\Delta <0$ shows the band offset. We must stress that an important step towards fully understanding possible strongly correlated phases relies on an effective model as explained in the effective tight-binding Hamiltonian.

The hopping matrix elements of the effective tight-binding model were obtained in the Wannier basis for the top two bands as a function of $\theta$ in Ref.^49^. The effective tight-binding Hamiltonian ($H_{\textrm{ETB}}$) was then constructed as:

9 $$\begin{aligned} H_{\textrm{ETB}}=\ t_1\sum _{<i,j>,\sigma } c_{i\sigma }^\dag c_{j\sigma } + \left| t_2\right| \ \sum _{\ll i,j\gg ,\sigma }{e^{i\phi \sigma \nu _{ij}}c}_{i\sigma }^\dag \ c_{j\sigma }+\cdots \end{aligned}$$

where $c_{j\sigma }$ and $c_{i\sigma }^\dag$ are fermionic annihilation/creation operators; $\sigma = \pm$ represents the spin/valley degree of freedom, the $\sum _{<i, j>}$ is over nearest neighbouring sites *i* and *j* (while $\sum _{\ll i,j\gg }$ for next nearest neighbouring sites), and $\nu _{ij} = \pm 1$ shows the path $i \rightarrow j$ turns right ($+$) or left (−).

The parameter $t_1$ is the real hopping term, while $t_2 = \left| t_2\right| e^{i\Phi }$ is the complex hopping term. Devakul et al.^49^ found that $\left| t_n\right|$ with hopping distance n reduce in magnitude, and only the imaginary component of $t_2$ has significant magnitude. The interlayer distance of hetero-bilayers of WS_2_/MoS_2_ and WSe_2_/MoSe_2_ and their unitcell constants were taken from the Ref.^50^ and represented in Table 1.

**Table 1:** Structural parameters of the studies hetero-bilayers.

Hetero-bilayer	*a* (nm)	*d* (nm)
WS_2_/MoS_2_	0.319	0.615
WSe_2_/MoSe_2_	0.332	0.647

*a* is the unitcell constant and *d* is the interlayer distance, both in units of nm.

Similar to twisted bilayer graphene ^1,13,51^, twisted heterostructures of bilayer TMD nanoribbons with flat bands near the Fermi level are good candidates for studying the strongly correlated phases.

Fang et al.^52^ calculated the band structure of untwisted TMD bilayers by using Slater-Koster expressions for the interlayer hopping interaction between chalcogen *p*-orbitals. They also included the value of the interlayer hopping between transition metal $d_{z_2}$-orbitals and chalcogen $p_z$-orbitals without using a Slater-Koster expression for the specific geometry of an untwisted 2*H* bilayer^52^. For twisted TMD bilayers, we used the Slater-Koster formula for generalizing the description of $p_z$ to $d_{z_2}$ hopping interactions as^27^:

10 $$\begin{aligned} t_{p_z,d_{z^2}}\left( r\right){} & {} =\ n\left[ n^2-\ \frac{1}{2}\ \left( l^2+\ m^2\right) \right] V_{pd\sigma }\left( r\right) \nonumber \\{} & {} \quad +\sqrt{3}n\ \ \left( l^2+\ m^2\right) V_{pd\pi }\left( r\right) , \end{aligned}$$

where the $l = r_x/r$, $m = r_y/r$ and $n = r_z/r$ were directional cosines. Functions of $V_{pd\sigma }\left( r\right)$ and $V_{pd\pi }\left( r\right)$ were obtained from Ref.^27^ by using a Wannier transformation of the DFT Hamiltonian.

Finally, we included spin-orbit coupling interactions, ${\hat{H}}_{\textrm{SOC}}=\frac{\lambda _{\textrm{SOC}}}{{2}}\ {{\hat{\tau }}}_y{{\hat{\sigma }}}_z$, where the orbital degree of freedom is represented via Pauli matrices ${\hat{\tau }}_y$ and ${\hat{\sigma }}_z$ acts on the spin, lifts the orbital degeneracy and opens up a gap at the $\Upsilon$ point.

The spin-orbit coupling in monolayers and moiré systems can lead to various Hall phenomena. For instance, spin-orbit coupling is responsible for the conversion of the orbital Hall effect to spin Hall effect in monolayers TMDs^53–57^. Moreover, other studies have found that the inclusion of spin-orbit coupling plays a key role in the observation of the moiré flat bands and correlated quantum anomalous Hall states in homo-bilayer MoS_2_ systems ^58^.

### Spectral properties of TMD nanoribbons with non-trivial band topology

We adopted a simulation method for calculating the spectral properties and response functions of heterostructure TMD nanoribbons with non-trivial band topology^59,60^.

We analyzed the quantum anomalous Hall conducting regime of electrified TMD hetero-nanoribbons with interfacial broken inversion symmetry incorporating SOC, which induced electric exchange ($\delta _{ex}$) as a scalar disorder^61,62^, where Hamiltonian is given by:

11 $$\begin{aligned} {\hat{H}}=\ -t\ \sum _{<ij>,s}{{{{\hat{c}}}_{is}}^\dag {{\hat{c}}}_{js}+\ \frac{2i}{3}}\ \sum _{<i,j>,s,s'}{{{{\hat{c}}_{is}}^\dag {{\hat{c}}}_{js}}}\left( \lambda _R \left( s \times d_{ij}\right) _z\right) _{ss'} + \delta _{ex} \sum _{i,s}{{{\hat{c}}}_{is}}^\dag {\hat{s}}_z {{\hat{c}}}_{js}. \end{aligned}$$

Here, the parameter *t* in the first term is the nearest-neighbour hopping term, and ${{{\hat{c}}}_{is}}^\dag$ $({{\hat{c}}}_{js})$ is the creation (annihilation) operator which adds (removes) electrons with the spin state $s =\, \uparrow , \downarrow$ to site *i*. The second term describes the Bychkov-Rashba spin-orbit coupling (BRSOC) with coupling strength $\lambda _R$, where ${\hat{s}}$ is a vector of Pauli matrices and the $d_{ij}$ acts as the unit vector pointing from the site *j* to *i*. The exchange field induced by the electric field is described in the last term with strength $\delta _{ex}$. The exchange field breaks the time-reversal symmetry and elastic back-scatterings at the edges are protected by spin-polarized. Consequently, the anomalous Hall conductivity has quantized form of $\sigma _{xy} = 2e^2/h$.

### Linear and non-linear conductivity tensors

In what follows, we present the calculation steps of electronic response functions (such as electrical conductivity) of a TB model subjected to an external electric field $E(t) = -\partial _t A(t)$^63^. The electrical current operator was calculated from the Hamiltonian using

12 $$\begin{aligned} {{\hat{J}}}^{\alpha \ }=\ -{\Omega }^{-1}\ \partial H/\partial A^\alpha , \end{aligned}$$

where $\Omega$ is the volume and $\alpha = x, y, z$ stands for the spatial direction. The operator takes the form

13 $$\begin{aligned} {{\hat{J}}}^\alpha (t){} & {} =-\ \frac{e}{\Omega }\ ({{\hat{h}}}^\alpha +e\ {{\hat{h}}}^{\alpha \beta }A^\beta \left( t\right) \nonumber \\{} & {} \quad +\frac{e^2}{2!}{{\hat{h}}}^{\alpha \beta \gamma }A^\beta \left( t\right) A^\gamma \left( t\right) +\cdots ), \end{aligned}$$

where ${{\hat{h}}}^{\alpha }$ is the single-particle velocity operator and defined as^63^:

14 $$\begin{aligned} {{\hat{h}}}^{\alpha _1\ldots ..\alpha _n}=\ \frac{1}{{(i\hbar )}^n}\ [{{\hat{r}}}^{\alpha _1},\ [\ldots [{{\hat{r}}}^{\alpha _n},\ {\hat{H}}]]], \end{aligned}$$

with ${\hat{r}}$ represents the position operator. The conductivity tensor $\sigma ^{\alpha \beta }$ can be described as^63^:

15 $$\begin{aligned} \sigma ^{\alpha \beta }\left( \omega \right) =\ \frac{ie^2}{\Omega \omega }\ \int _{-\infty }^{\infty } d\epsilon \ f(\epsilon ) \ Tr\left( {{\hat{h}}}^{\alpha \beta } \ \delta (\epsilon - {\hat{H}}) + \frac{1}{\hbar } {{\hat{h}}}^{\alpha } g^R (\epsilon + \hbar \omega ) \ {{\hat{h}}}^{\beta } \delta (\epsilon - {\hat{H}})+ \frac{1}{\hbar } {{\hat{h}}}^{\alpha } \delta (\epsilon - {\hat{H}}) \ {{\hat{h}}}^{\beta } g^A (\epsilon - \hbar \omega )\right) , \end{aligned}$$

and the non-symmetrized second-order conductivity $(\sigma ^{\alpha \beta \gamma })$ would be:

16 $$\begin{aligned} \sigma ^{\alpha \beta \gamma }\left( \omega _1,\ \omega _2\right){} & {} =\ \frac{1}{\Omega }\frac{e^3}{\omega _1\omega _2}\ \int _{-\infty }^{\infty }d\epsilon \ f\left( \epsilon \right) Tr[ {\frac{1}{2}{\hat{h}}}^{\alpha \beta \gamma }\delta \left( \epsilon -\ {\hat{H}}\right) \ + \frac{1}{\hbar } {{\hat{h}}}^{\alpha \beta }\ g^R\ \left( 
\epsilon +\ \hbar \omega _2\right) \ {{\hat{h}}}^\gamma \ \delta \left( \epsilon -\ {\hat{H}}\right) \nonumber \\{} & {} \quad +\ \ \frac{1}{\hbar }\ {{\hat{h}}}^{\alpha \beta }\ \delta \left( \epsilon -\ {\hat{H}}\right) \ {{\hat{h}}}^\gamma \ g^A\ \left( \epsilon -\ \hbar \omega _2\right) ] +\frac{1}{2\hbar }\ {{\hat{h}}}^\alpha \ g^R\ \left( \epsilon +\hbar \omega _1+\ \hbar \omega _2\right) \ {{\hat{h}}}^{\beta \gamma }\ \delta \left( \epsilon -\ {\hat{H}}\right) \nonumber \\{} & {} \quad +\frac{1}{2\hbar }\ {{\hat{h}}}^\alpha \delta \left( \epsilon -\ {\hat{H}}\right) {{\hat{h}}}^{\beta \gamma }\ g^A\ \left( \epsilon -\hbar \omega _1-\ \hbar \omega _2\right) +\frac{1}{\hbar ^2}\ {{\hat{h}}}^\alpha \ g^R\ \left( \epsilon +\hbar \omega _1+\ \hbar \omega _2\right) \ {{\hat{h}}}^\beta \ g^R\ \left( \epsilon +\ \hbar \omega _2\right) \ {{\hat{h}}}^\gamma \ \delta \left( \epsilon -\ {\hat{H}}\right) \nonumber \\{} & {} \quad +\ \ \frac{1}{\hbar ^2}\ {{\hat{h}}}^\alpha \ g^R\ \left( \epsilon +\hbar \omega _1\right) \ {{\hat{h}}}^\beta \ \delta \left( \epsilon -\ {\hat{H}}\right) \ {{\hat{h}}}^\gamma \ g^A\ \left( \epsilon -\ \hbar \omega _2\right) \nonumber \\{} & {} \quad +\frac{1}{\hbar ^2}\ {{\hat{h}}}^\alpha \delta \left( \epsilon -\ {\hat{H}}\right) {{\hat{h}}}^\beta \ g^A\ \left( \epsilon -\hbar \omega _1\right) \ {{\hat{h}}}^\gamma \ g^A\ \left( \epsilon -\ \hbar \omega _1-\ \hbar \omega _2\ \right) . \end{aligned}$$

### Electron–electron interactions

The electron–electron (e–e) interaction strongly depends on the e−e scattering length ($l_{ee}$), which is tunable by temperature and charge density. To describe interacting electrons as interacting fermions, the Fermi liquid theory determines that e−e mean free paths ($l_{ee}$) can be calculated from $l_{ee} = \nu _{\textrm{F}} \tau _{ee}$ where $\tau _{ee}\approx \ \frac{\hbar E_{\textrm{F}}}{(K_{\textrm{B}}T)^2}$; $\nu _{\textrm{F}}$ is Fermi velocity and 1/$\tau _{ee}$ is the e−e scattering rate. Therefore, for a small temperature near zero, the scattering rate goes to zero, and then ignoring e−e interactions for a small temperature can be justified reasonably. Moreover, to investigate the electron interaction of hetero-layers, we construct several defective and strained heterostructures, in which interlayer electron states generate flat bands, split topological bands, and open a fascinating route to achieve tunable terahertz quantum devices.

**Coulomb interaction and valley polarized:** The Coulomb interaction due to the spin-valley locking in moiré TMD hetero-nanoribbons defines as^30^:

17 $$\begin{aligned} H_{\textrm{interaction}}{} & {} = \frac{1}{2S} \sum _{k,k',q} V(q)c^\dagger_{\tau }(k + q)\nonumber \\{} & {} \quad c^\dagger _{\tau '}(k' - q) c_{\tau '}(k')c_\tau (k), \end{aligned}$$

where *S* is the sample area, and $V(q) = \frac{e^2}{2\epsilon \epsilon _0} \sqrt{q^2 + k^2}$ is the screened Coulomb interaction with $\lambda ^{-1}$ denoting a screened length and $\epsilon$ and $\epsilon _0$ denoting the dielectric constant and vacuum permittivity, respectively. The spin-valley locking in TMD hetero-nanoribbons leads to correlated ground states, classified as half-filling states; where the spin-valley-polarized (SVP) state

$$\begin{aligned} |\psi _G> = \Pi _{|k|<k_F} c\dagger _\tau |0>, \end{aligned}$$

and only $\tau$-valley is occupied^30^. The SVP at half-filling state breaks time-reversal symmetry, and lifts the valley degeneracy, leading to the valley-polarized quantum anomalous Hall insulating state.

### Hamiltonian of strained lattices

**Figure 2:**
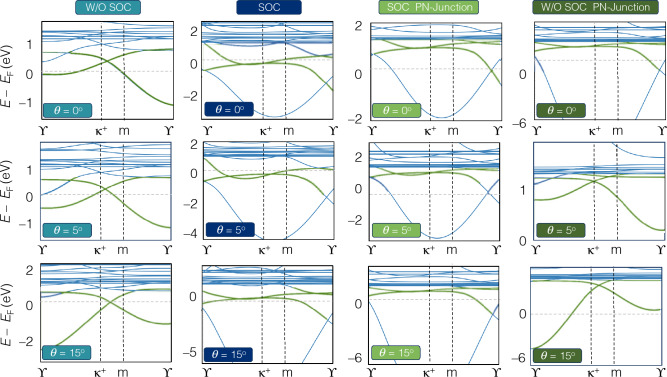
Band structure of rectangular armchair hetero-nanoribbons WS_2_/MoS_2_ for twist angles ($\theta$ = 0^∘^, 5^∘^, 15^∘^) without and with spin-orbit coupling and PN junction (electric field) along *z*-axis of hetero-nanoribbons (1 eV/nm), (see Figs. S1–S3 for more details).

Following the method we applied in our previous study on strain engineering of TMD nanoribbons, we utilized the Slater-Koster TB approach for lattice deformations, where the effect of strain is captured by varying TB hopping parameters depending on the inter-atomic bond length, as illustrated in Ref. ^64^. Hence, the strain field modifies the hopping terms and can be written as:

18 $$\begin{aligned} t_{ij,\mu \nu }=\ t_{ij,\mu \nu }\left( r_{ij}^0\right) \left( 1-\ \Lambda _{ij,\mu \nu }\frac{|r_{ij}-\ r_{ij}^0|}{|r_{ij}^0|}\right) , \end{aligned}$$

where $|r_{ij}^0|$ is the atomic distance of the unstrained position of two atoms of $(i,\mu )$ and $(j,\nu )$ at the equilibrium positions, while $|r_{ij}|$ is the atomic distance in the strained structure. In Eq. (18),

19 $$\begin{aligned} \Lambda _{ij,\mu \nu }=\ \frac{-d\ ln(t_{ij,\mu \nu })}{d\ ln\left( r\right) |_{r=|r_{ij}^0|}}, \end{aligned}$$

is the local electron-phonon coupling, and the Wills-Harrison argument as $t_{ij,\mu \nu }\left( r\right) \propto \ {|r|}^{-(l_\mu +\ l_\nu +1)}$ were used due to the absence of any theoretical and experimental data for the electron-phonon coupling, where $l_{\mu (\nu )}$ is the absolute value of the angular momentum of orbital $\mu (\nu )$. Furthermore, $\left| r_{ij}^0\right| =\ \sqrt{\frac{7}{12}}a$ for M-X bond and $\left| r_{ij}^0\right| =\ a$ for in-plane M-M bond have been applied^64^. Following this approach, $\Lambda _{ij,\mathrm M-M}=5$for M-M dd and $\Lambda _{ij,\mathrm X-X}=3$ for X-X pp hybridization take into account, where $\Lambda _{ij,\mathrm X-M}=4$ for X-M pd hybridization has been applied^64^. The symmetric strain tensor for 2D materials defines as^64^:

20 $$\begin{aligned} \epsilon =\ \left( \begin{matrix}\varepsilon _{xx}&{}\varepsilon _{xy}\\ \varepsilon _{xy}&{}\varepsilon _{yy}\\ \end{matrix}\right) \end{aligned}$$

where components of strain tensor ($\varepsilon _{ij}$) include the in-plane ($u_{ii}$) and the out-of-plane ($u_{ij}$) displacement as:

21 $$\begin{aligned} \varepsilon _{ij}=\ \frac{1}{2}\ \left( \frac{{\partial u}_i}{{\partial r}_j}+\ \frac{{\partial u}_j}{{\partial r}_i}\right) +\ \frac{1}{2}\ \frac{{\partial u}_z}{{\partial r}_i}\ \frac{{\partial u}_z}{{\partial r}_j}, \end{aligned}$$

where $r = (x,y)$ is the position vector and $u = (u_x , u_y , u_z)$ is the displacement vector. Moreover, the local anti-symmetric rotation tensor ($\omega$) in the system defined as:

22 $$\begin{aligned} 2 \omega _{xy} = -2 \omega _{yx} = \left( \frac{{\partial u}_y}{\partial x}+\ \frac{{\partial u}_x}{\partial y}\right) , \end{aligned}$$

and for homogeneous strain rotation tensor will be zero. When applying strain field in the system, we considered the transformation relation of $r \propto r_{\textbf{0}}+ r_{\textbf{0}}\cdot \mathbf {\nabla }u$ for electron hopping terms, where $u=\varepsilon +\ \omega$^64^.

## Results and discussion

A moiré pattern can be formed by stacking 2*H* layered group VI TMDs such as MoS_2_ and WS_2_. However, when group IV 1*T* TMDs such as ZrS_2_ are interfaced, twisting them would lead to a Kagome lattice. Monolayer TMDs such as MoTe_2_ and WSe_2_ exhibit spin-valley locking, *i*.*e*., top valence bands with spin $\downarrow$ at −**K** and spin $\uparrow$ at $+$ **K**^65^. In our study, **K** points of twisted hetero-TMD nanoribbons were displaced and formed the $\kappa ^{+}$ and $\kappa ^{-}$ at the corners of the moiré Brillouin zone. Hybridized $+$ **K** (−**K**) valley bands of the two nanoribbons create a set of spin $\uparrow$ ($\downarrow$) moiré bands as shown in Fig. 1.

### Electronic band structure, spin-orbit coupling and PN-junction (electric field)

**Figure 3:**
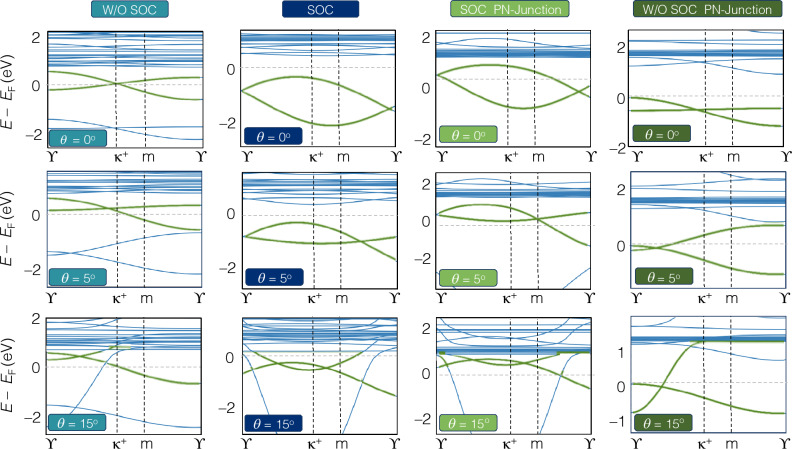
Band structure of rectangular armchair hetero-nanoribbons WSe_2_/MoSe_2_ for twist angles ($\theta$ = 0^∘^, 5^∘^, 15^∘^) without and with spin-orbit coupling and PN junction (electric field) along *z*-axis of hetero-nanoribbons (1 eV/nm), (see Figs. S1–S3 for more details).

**Figure 4:**
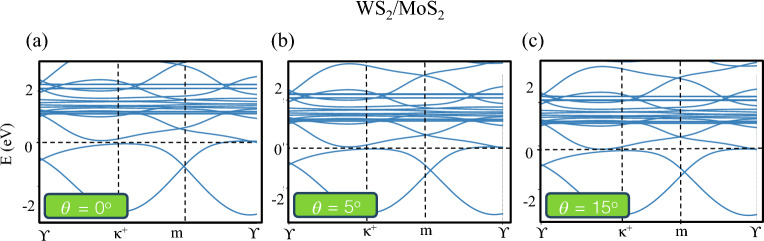
The band structure of WS_2_/MoS_2_ by considering the Coulomb interaction and spin-orbit coupling (SOC) in the Hamiltonian Eq. (17).

To investigate the electronic properties of hetero-TMD nanoribbons, their electronic band structures via TB-Hamiltonian discussed in Eq. (9) were calculated with and without SOC as well as PN-junction along the *z*-axis of hetero-nanoribbons. 2D TMD structures of group VI and their bilayers are semiconductors and their twisted homo/hetero-bilayers exhibit different behaviours depending on twisted angles. Herein, all armchair transition metal sulfide hetero-nanoribbons, AC-WS_2_/MoS_2_, display a gap-less topological nature at $\kappa ^{+}$ point in the absence of SOC. The flat Dirac cones are shifted towards $\Upsilon$ point upon applying SOC (Figs. 2, S1–S3), and flat bands emerge as a signature of topological bands.

**Figure 5:**
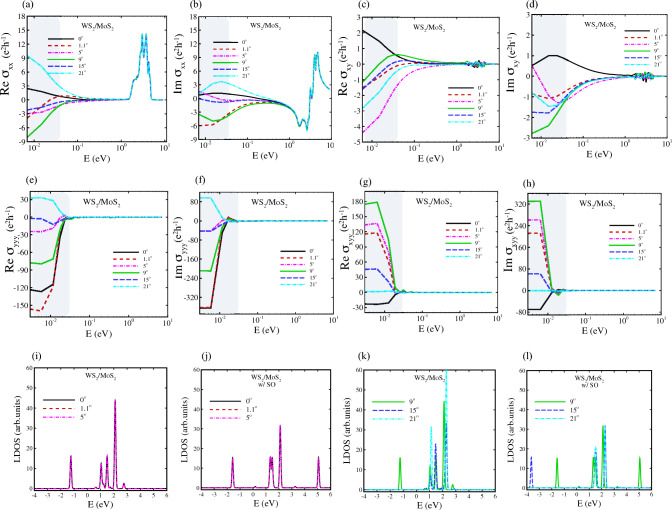
(**a**–**d**) Quantum Hall effect of armchair hetero-nanoribbons WS2/MoS2 for rotation angles of $\theta = 0^\circ , 1.1^\circ , 5^\circ , 9^\circ , 15^\circ , 21^\circ$. The top panels display the real and imaginary part of longitudinal conductivity $\sigma _{\textrm{xx}}$ and the Hall conductivity $\sigma _{\textrm{xy}}$. The gray region shows the terahertz region. (**e**–**h**) Non-linear quantum Hall effect of armchair hetero-nanoribbons WS_2_/MoS_2_ for the same twisted angles of top panels. (**e**) and (**f**) display the real and imaginary part of longitudinal conductivity $\sigma _{\textrm{yyy}}$ and (**g**) and (**h**) are for Hall conductivity $\sigma _{\textrm{xyy}}$. The gray region shows the terahertz region. (**i**–**l**) Local density of states of armchair WS_2_/MoS_2_ hetero-nanoribbons at the same twisted angles.

**Figure 6:**
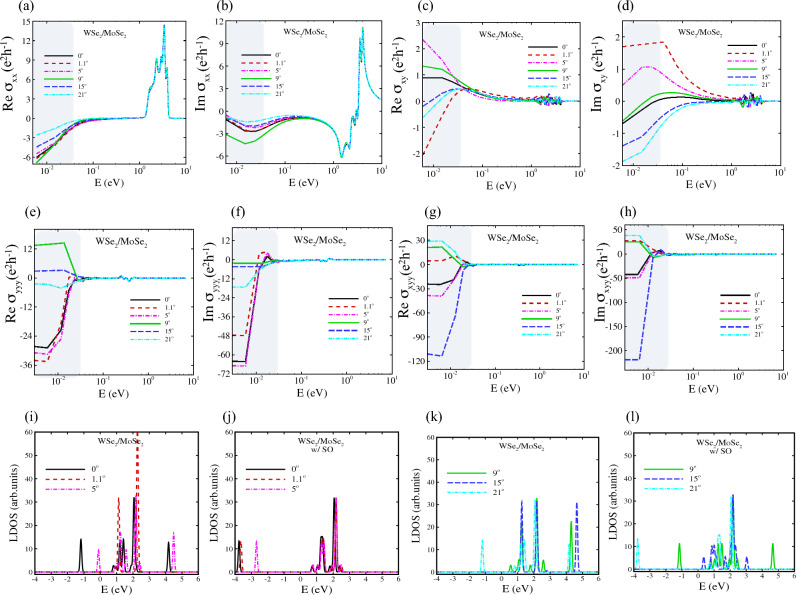
(**a**–**d**) Quantum Hall effect of armchair hetero-nanoribbons WSe2/MoSe2 for rotation angles of $\theta = 0^\circ , 1.1^\circ , 5^\circ , 9^\circ , 15^\circ , 21^\circ$. The top panels display the real and imaginary part of longitudinal conductivity $\sigma _{\textrm{xx}}$ and the Hall conductivity $\sigma _{\textrm{xy}}$. The gray region shows the terahertz region. (**e**–**h**) Non-linear quantum Hall effect of armchair hetero-nanoribbons WSe_2_/MoSe_2_ for the same twisted angles of top panels. (**e**) and (**f**) display the real and imaginary part of longitudinal conductivity $\sigma _{\textrm{yyy}}$ and (**g**) and (**h**) are for Hall conductivity $\sigma _{\textrm{xyy}}$. The gray region shows the terahertz region. (**i**–**l**) Local density of states of armchair WSe_2_/MoSe_2_ hetero-nanoribbons at the same twisted angles.

**Figure 7:**
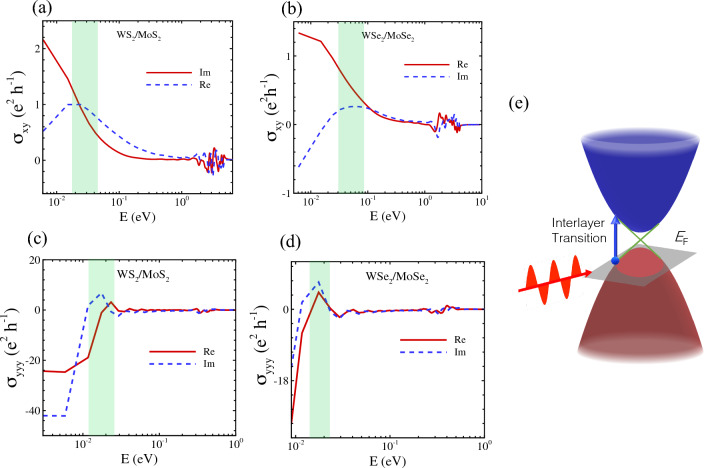
(**a**, **b**) The Hall conductivity of TMD hetero-nanoribbons, where the green filled area indicates the resonance Hall conductivity due to the vertical transition of fermions. (**c**, **d**) The same as part (**a**, **b**) for nonlinear Hall conductivity of WS_2_/Mo$S_2$ and WSe_2_/MoSe_2_. (**e**) A schematic model of vertical transition in Hall conductivity of topological materials.

When selenium is substituted as the chalcogen atom, *i*, *e*. in AC-WSe_2_/MoSe_2_, strong SOC causes band splitting as shown in Figs. 3 and S4–S6. The SOC lifts the conduction band minimum with the downshifted valence band maximum for some twisted angles, where SOC induces band gap opening at the Fermi level and is an indicator for a topologically non-trivial material.

**Figure 8:**
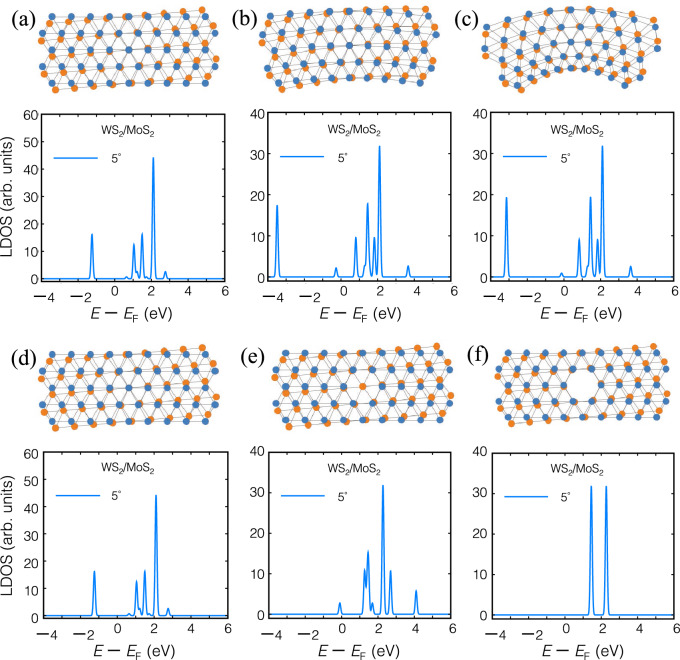
Uniaxial Y-arc strain of twisted WS_2_/MoS_2_ hetero-nanoribbons with a rotation angle $\theta = 5^{\circ }$. (**b**, **c**) Local density of states (LDOS) of Y-arc strain field of $C = 0.05$ and 0.2 are compared with unstrained twisted heteronanoribbons (**a**). Vacancy defect of twisted WS_2_/MoS_2_ hetero-nanoribbons with a rotation angle $\theta = 5^{\circ }$. (**e**, **f**) LDOS of monolayer and bilayer vacancy defect are compared with pristine twisted hetero-nanoribbons (**d**).

Upon inclusion of SOC, AC-WSe_2_/MoSe_2_ hetero-nanoribbons exhibit three twist angle-dependent regimes. Hetero-nanoribbons (1) with zero twist angle show an indirect band gap, (2) with $\theta < 9^{\circ }$ show a direct band gap, and (3) with $\theta \ge 9^{\circ }$ indicate gapless or topological behaviour.

**Figure 9:**
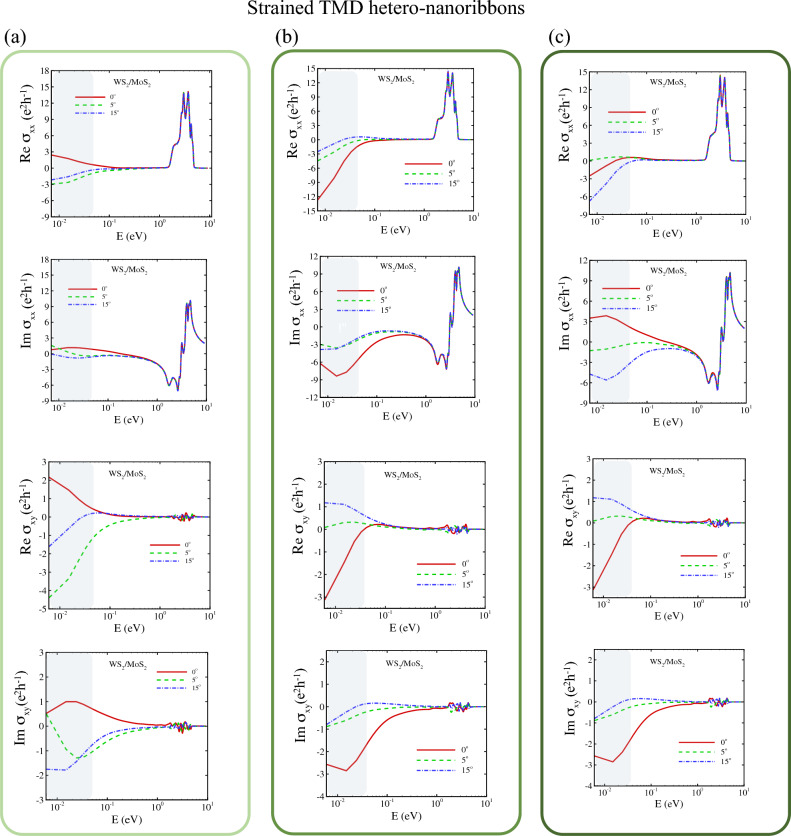
Quantum Hall effect of uniaxial Y-arc strained twisted WS_2_/MoS_2_ hetero-nanoribbons. The real and imaginary part of longitudinal conductivity $\sigma _{xx}$ and the Hall conductivity $\sigma _{xy}$ are shown for (**a**) pristine, (**b**) $C = 0.05$ and (**c**) $C = 0.2$ strain fields. Gray region indicates the terahertz range.

**Figure 10:**
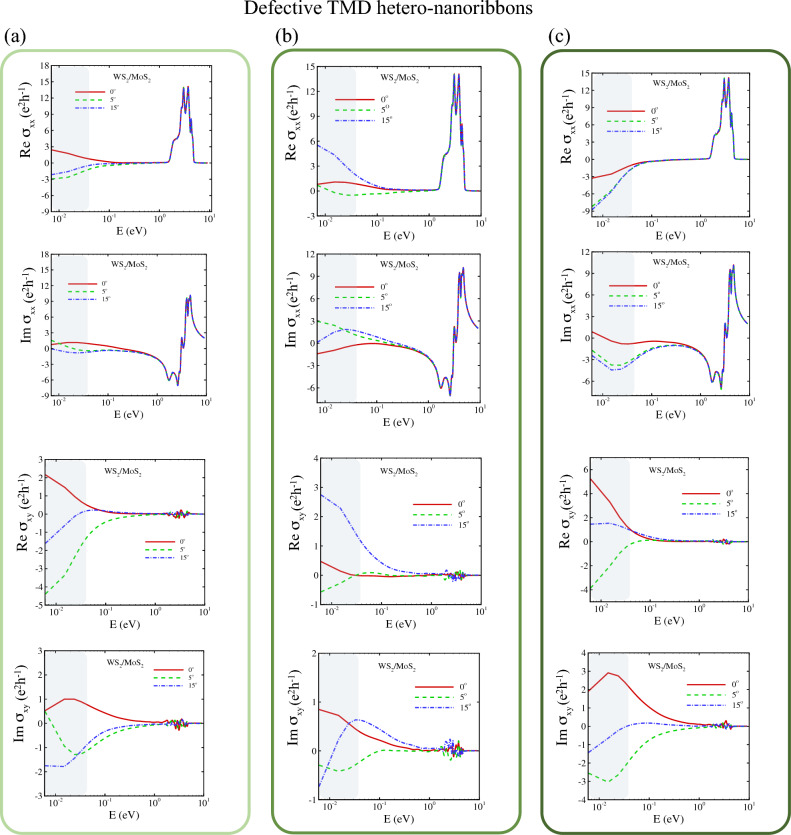
Quantum Hall effect of twisted WS_2_/MoS_2_ hetero-nanoribbons with vacancy defects. The real and imaginary part of longitudinal conductivity $\sigma _{xx}$ and the Hall conductivity $\sigma _{xy}$ are shown for (**a**) pristine, (**b**) monolayer and (**c**) bilayer vacancy defects as depicted in Fig. 8. Gray region indicates the terahertz range.

The effect of forming PN-junctions (1 eV/nm) on the electronic band structure of hetero-nanoribbons with SOC and without SOC is shown in Figs. 2 and 3 (see Figs. S1–S6 for more details).

To apply perpendicular electric field in TMD hetero-nanoribbons, a P-N junction is used as a vertical boundary or interface in z axis of hetero-nanoribbons, which divides our structures to positive potential energy as p-type and negative potential energy as n-type.

The inclusion of SOC, causes an uplift of band energies besides creating flat bands in the conduction zone for twisted hetero-nanoribbons of both types. The electronic and topological properties of all ZZ-TMD hetero-nanoribbons, including their electronic band structures, are calculated and shown in the Supporting Information (Figs. S7–S12).

The emergence of a non-trivial band topology due to a large Dirac cone at the **K** point without band inversion has been shown previously in other materials such as BiX (X=H, F, Cl and Br) structures^66^. Furthermore, the flat bands of the strongly correlated regime of TMD hetero-nanoribbons create a spin/valley polarization, which causes the quantum anomalous Hall (QAH) insulator state, as SOC in TB Hamiltonian conserves the *z*-spin/valley component.

### Topological insulating states

The intrinsic SOC can cause topological states in moiré superlattices. Good examples are twisted bilayer^9,10,67^ and trilayer graphene^68^. In addition, the interplay of valley polarization, ferromagnetic order and substrate engineering effects can also induce the non-trivial band topology. For instance, the anomalous quantum Hall effect in MoTe_2_/WSe_2_^29^ emerges from spontaneous valley polarization and can be generalized to other twisted transition-metal dichalcogenides^30,49^.

Our results reveal that moiré bands of twisted AC-WS_2_/MoS_2_ nanoribbons can be topologically non-trivial by considering valence band states in $\kappa ^{+}$ valleys, as shown in Fig. 2. Moreover, the dispersionless conduction moiré bands realize a topological quantum spin Hall insulator with a spin Chern number. Note that studied hetero-nanoribbons of both sulfide and selenide types, exhibit flat topological moiré bands which are highly tunable with SOC.

Zhang et al. ^28^ in their work indicated that the Coulomb interaction only renormalizes the band structure by the mean-field Hatree potential approximation in agreement with previous works^69,70^. As conclusion, the Coulomb interaction in moiré TMD hetero-nanoribbons lifts the valley degeneracy as shown in Fig. 4.

In this work, we propose that lattice relaxation in moiré TMD hetero-nanoribbons induce the pseudo-magnetic fields, which could give rise to topologically nontrivial moiré bands with finite Chern numbers. We show that Coulomb interaction at half-filling $\nu = 1$ lifts the degeneracy of the two valleys as plotted in Fig. 4 and results in a valley-polarized quantum anomalous Hall state, as observed in the experiment^30^.

### Linear anomalous Hall effect

The experimental realization of enhanced electromagnetic phenomena due to geometrical electronic states creates the giant anomalous Hall effect (AHE) in topological semimetals. Using THz and infrared magneto-optical spectroscopy, Kato et al.^71^ showed the existence of two significant resonance structures in the optical Hall conductivity spectra $\sigma _{xy}(\omega )$, related to the AHE.

Our calculated local density of states (LDOS) for TMD hetero-nanoribbons indicate large DOS values corresponding to the presence of flat bands. Moreover, band crossings near the Fermi level cause large AHC values, consistent with the findings of Kato et al.^71^.

In this section, we calculate and plot optical response functions ${\hat{\sigma }}(\omega )$ (real and imaginary parts), for heterostructures of WS_2_/MoS_2_ and WSe_2_/MoSe_2_ nanoribbons by providing the framework for tackling multi orbital TB models in the presence of disorder as external electric fields. Figures 5a–d and 6a–d show the real part and imaginary part of $\sigma _{xx}(\omega )$ and $\sigma _{xy}(\omega )$ (Eq. 15) at low frequencies (DC regime) as highlighted by gray colour. However, the real part of quantum conductivity has significant value in other regions. The large AHE is attributed to not only the Berry curvature on the band crossings near the Fermi level but also flat bands in conduction bands cooperatively produce the large intrinsic AHE in nodal line of AC-WS_2_/MoS_2_ as represented in Fig. 5a–d.

### THz properties and non-linear anomalous Hall effect

The electromagnetic spectrum between microwave and infrared waves is known as terahertz (THz) radiation. 2D materials such as black phosphorus (BP), graphene and TMDs are known to be able to propagate THz waves. Moreover, artificially engineered meta-materials combined with 2D materials could modulate and manipulate THz radiation for novel terahertz applications^72–76^.

TMD structures have been previously studied as candidates for THz applications^77–79^. Note that the difference in space group symmetry of 2D materials can lead to different optical behaviours. For instance, the graphene lattice with D_6h_ point group as a centrosymmetric material, forbids second harmonic generation (SHG) and a non-linear optical process^80^. On the contrary, the strong process of third-harmonic generation (THG) in graphene makes it a suitable material for non-linear optical applications^81,82^. The lowest-order non-linear optical process of TMDs is SHG as a consequence of the D_3h_ point group of TMD monolayers.

In this study, we investigate the THz conductivity modulation based on the non-linear Hall effect of TMD hetero-nanoribbons by using the non-linear surface conductivity tensor of Eq. (16). The computational results of the second-order conductivity of moiré hetero-nanoribbons of both types are plotted in Figs. 5e–h and 6e–h, where the THz zones are highlighted in grey.

Midgap states of TMD semiconductors affect their transport properties, which are induced by native or intentionally incorporated defects, dopants, electric fields and strain fields in the crystal lattice^64,83–85^. We previously showed that the strain field in strained TMD nanoribbons creates midgap states which can be observed in their local density of states (LDOS), consistent with STM experimental data^64^. Herein, we calculated LDOS to figure out the origin of enhanced Hall conductivity in TMD hetero-nanoribbons. As shown in Figs. 5i–l and 6i–l, the twist angle plays a key role in altering and tuning the midgap states and enhancing the linear and non-linear Hall conductivity. The intrinsic AHE as a prominent feature in the optical Hall conductivity spectra $\sigma _{xy}(\omega )$ causes the interband optical transition on the topological electronic structure, giving rise to the resonant structure in $\sigma _{xy}(\omega )$^86–90^. The vertical transition near the Dirac point as plotted in Fig. 7 leads to the resonance peak at the energy range [0.02–0.1 eV] for the linear Hall effect as shown with the green filled area, while for the nonlinear Hall effect the resonance peak is located in energy range [0.01–0.03 eV].

### Impact of defect and strain as pseudoelectric and pseudomagnetic field (Landau levels)

Strain can play a crucial role in the physics of twisted heterostructures. Recently, Zhang et al.^91^ experimentally reported the existence of correlated states in strained twisted bilayer graphene away from magic angle by using transport measurement. They showed that hetero-strain caused during heterostructure stacking creates flat bands, confirming the presence of correlated and topological states^91^. Moreover, Guo et al.^92^ studied defective vdW heterostructures and demonstrated that defects and moiré potentials can trap excitons in these interfaces.

Inhomogeneous strains in TMD nanoribbons can effectively induce pseudoelectric and pseudomagnetic fields and tune both the magnitude and sign of valley Hall conductivity (VHC). The pseudomagnetic field introduces multiple Landau levels in the LDOS of strained TMD nanoribbons, separated by band gaps^64^.

In Y-arc strained twisted TMD hetero-nanoribbons as shown in Fig. 9a–c, stretching the bonds shifts the **K** and **K**^′^ momentum of Dirac cones in reciprocal space by $\delta k$ from their unstrained points, which generates a pseudovector potential term of $eA/c$ and opposite signs of pseudomagnetic fields at the two valleys^64^. The Y-arc strain creates dense regions in the TMD nanoribbons and separates $\pm {\hat{z}}$ pseudomagnetic fields of pseudo spin up and down. As a consequence, we observe the reversal of pseudospins forms the valley-polarized states.

The electronic band structure of Y-arc strained and defective TMD hetero-nanoribbons in Figs. S16 and S17 reveal two sets of flat bands. One set is located in the conduction band, which stems from moiré potential and the second flat band is formed near the Fermi level, originating from strain fields and vacancy defects due to the correlation of electron states in vdW hetero-nanoribbons, in close agreement with the experimental result of Ref.^91^. Furthermore, LDOS of the Y-arc strained TMD hetero-nanoribbons as shown in Fig. 8b, c, indicates the emergence of a new quantized Landau level as a result of the creation of a pseudomagnetic field besides inhomogeneous strain.

Finally, the influence of vacancy monolayer and bilayer defect on electronic properties of twisted hetero-nanoribbons (shown in Fig. 8e, f) is investigated and their optical conductivity as quantum Hall effect is plotted in Figs. 9 and  10. The electronic band structures shown in Fig. S17 indicate the formation of multiple flat bands for both types of vacancies, confirming localized electron states and correlated quantum states. Optical conductivities of these defective hetero-nanoribbons are calculated in terms of the real and imaginary part of longitudinal conductivity $\sigma _{xx}$ and the Hall conductivity $\sigma _{xy}$. Comparison of the terahertz region (gray region) with pristine structure reveals that the interplay of the defect and moiré potential can manipulate the positive and negative conductivity of twisted hetero-nanoribbons in the terahertz region.

## Conclusion

The present study sheds light on the intrinsic AHE in twisted WS_2_/MoS_2_ and WSe_2_/MoSe_2_ hetero-nanoribbons. The large AHC values are attributed to two cooperatively distinct features observed in the electronic structure of these systems, namely (1) the band crossings near the Fermi level, and (2) large DOS of flat bands. Furthermore, our results indicate the importance of considering strain fields and vacancy defects when dealing with strongly correlated quantum states at twist angles away from the magic angle. In view of the ubiquitous presence of flat bands in these heterostructures, twisted TMD hetero-nanoribbons seem well suited for THz detection and rectification. We found that the electronic structure of these interfaces is highly sensitive to SOC strength and twist angles. The tunability of hetero-TMD-based moiré systems provides an ideal setting for observing topological insulator states and remarkably flat Chern bands, creating quantum anomalous Hall states at zero magnetic fields. Our findings suggest that engineered moiré superlattices of TMDs have the potential to become a component of future nanoelectronics and quantum information technologies and can find application in new quantum coherent electronic devices.

### Supplementary Information

Supplementary Figures.

## Data Availability

The datasets generated during and/or analyzed during the current study are available from the corresponding author on reasonable request.
