# Publisher Correction: Terahertz linear/non-linear anomalous Hall conductivity of moiré TMD hetero-nanoribbons as topological valleytronics materials

**DOI:** 10.1038/s41598-024-56021-5

**Published:** 2024-03-14

**Authors:** Farzaneh Shayeganfar, Ali Ramazani, Hamidreza Habibiyan, Mohammad Rafiee Diznab

**Affiliations:** 1 Department of Physics and Energy Engineering, Amirkabir University of Technology, Tehran, Iran; 2 Department of Mechanical Engineering, Massachusetts Institute of Technology, Cambridge, MA 02139 USA; 3 Department of Physics and Atmospheric Science, Dalhousie University, Halifax, Nova Scotia B3H 4R2 Canada

Correction to: *Scientific Reports* 10.1038/s41598-024-51721-4, published online 18 January 2024

The original version of this Article contained an error in the spelling of the author Farzaneh Shayeganfar which was incorrectly given as Farzaneh Shayeganfa.

The original Article has been corrected.

