# Supplementary material for: Terahertz linear/non-linear anomalous Hall conductivity of moiré TMD hetero-nanoribbons as topological valleytronics materials
Source: Sci Rep. 2024 Jan 18;14:1581. doi: 10.1038/s41598-024-51721-4 (PMC10796390; doi:10.1038/s41598-024-51721-4)
Supplement: Supplementary file 1 — Supplementary Figures. [file 41598_2024_51721_MOESM1_ESM.pdf]

Supplementary Material for: Terahertz Linear/Non-linear Anomalous Hall Conductivity of moiré TMD Hetero-Nanoribbons as Topological Valleytronics Materials

Farzaneh Shayeganfar,[*,†,‡] Ali Ramazani,[¶] Hamidreza Habibiyan,[†] and Mohammad Rafiee Diznab[§]

†*Department of Physics and Energy Engineering, Amirkabir University of Technology, Tehran*

‡*Department of Aerospace Engineering, University of Michigan, Ann Arbor, MI 48109, USA*

¶*Department of Mechanical Engineering, Massachusetts Institute of Technology, Cambridge, MA 02139, USA*

§*Department of Physics and Atmospheric Science, Dalhousie University, Halifax, Nova Scotia B3H 4R2, Canada*

E-mail: fshayega@umich.edu

AC WS_2/MoS_2

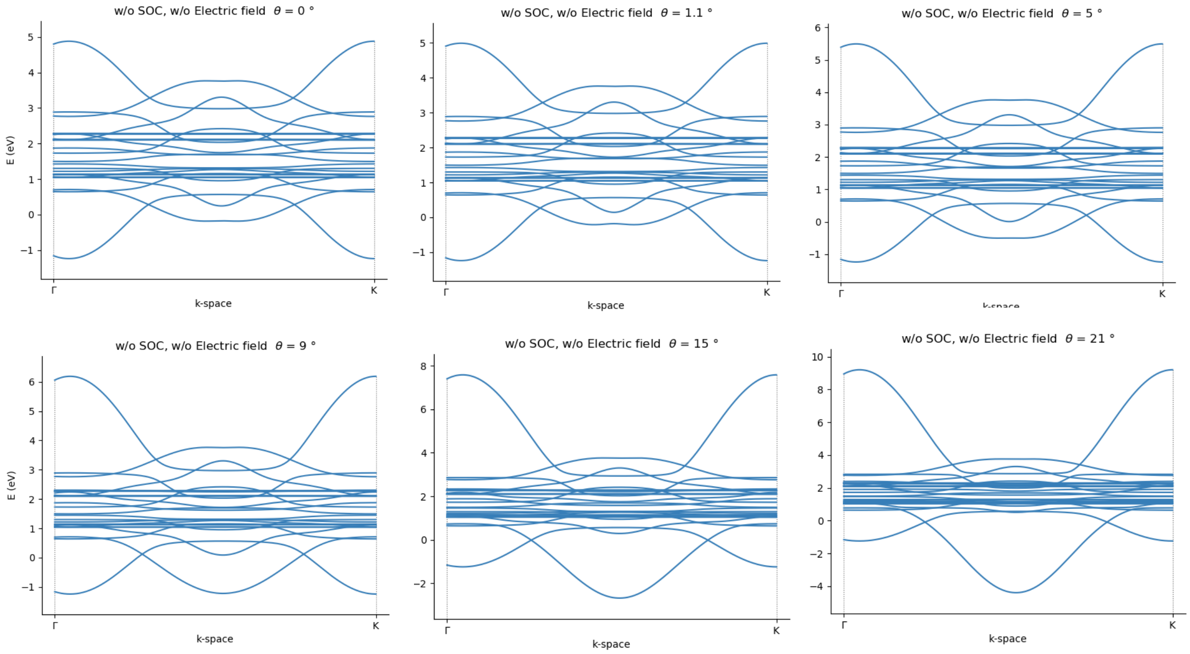

Fig. S 1: Band structure of rectangular armchair hetero-nanoribbons WS2/MoS2 for twist-angles ($\theta = 0°, 1.1°, 5°, 9°, 15°$, and $21°$) without spin-orbit coupling.

AC WS_2/MoS_2

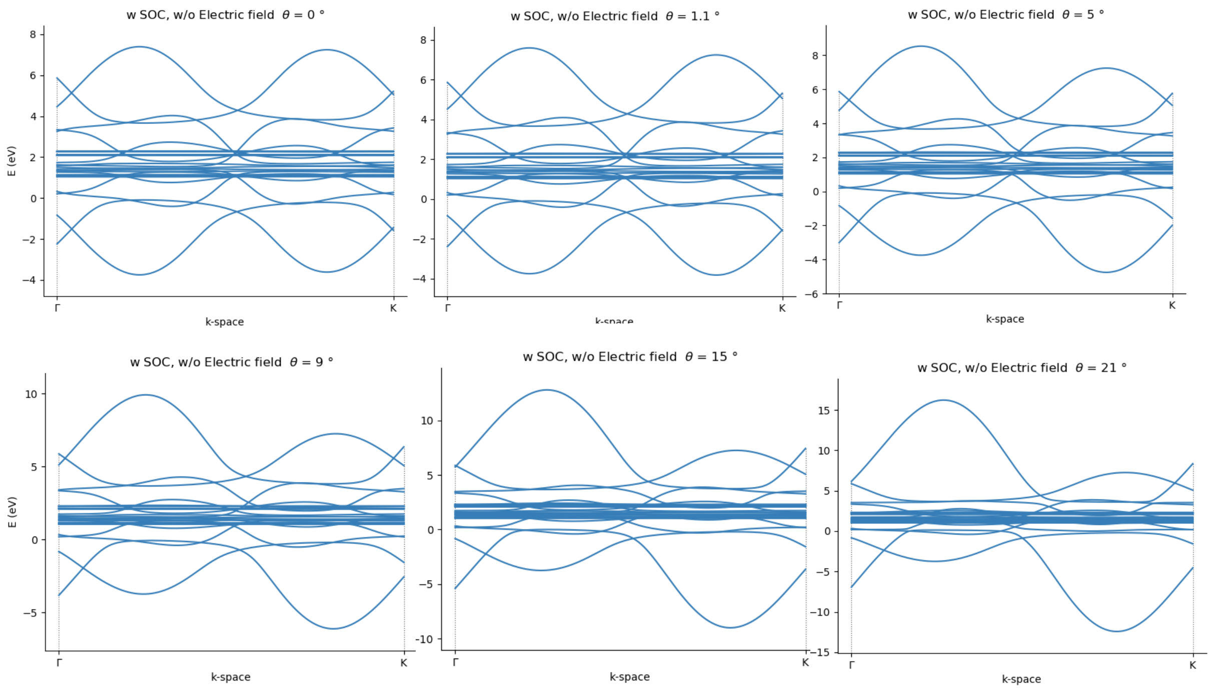

Fig. S 2: Band structure of rectangular armchair hetero-nanoribbons WS2/MoS2 for twist-angles ($\theta = 0°, 1.1°, 5°, 9°, 15°$, and $21°$) with spin-orbit coupling.

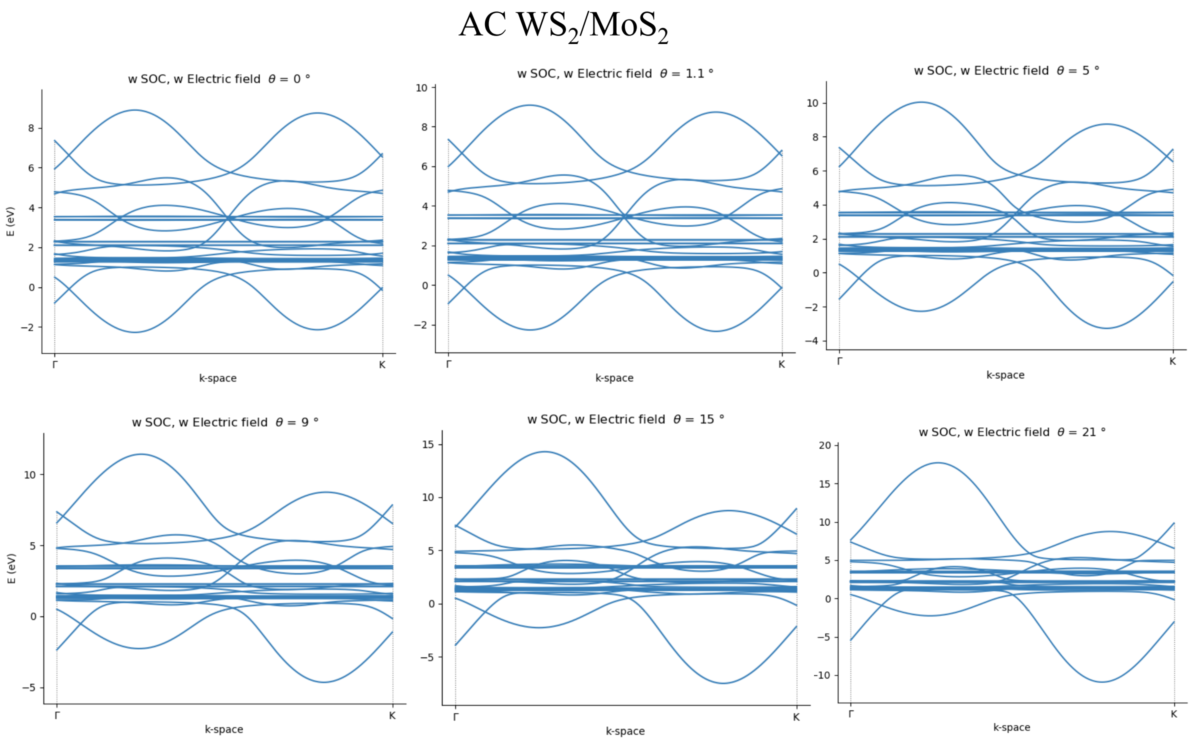

Fig. S 3: Band structure of rectangular armchair hetero-nanoribbons WS2/MoS2 for twist-angles ($\theta = 0°, 1.1°, 5°, 9°, 15°$, and $21°$) with spin-orbit coupling and with electric field (1 eV/nm).

AC $WSe_2/MoSe_2$

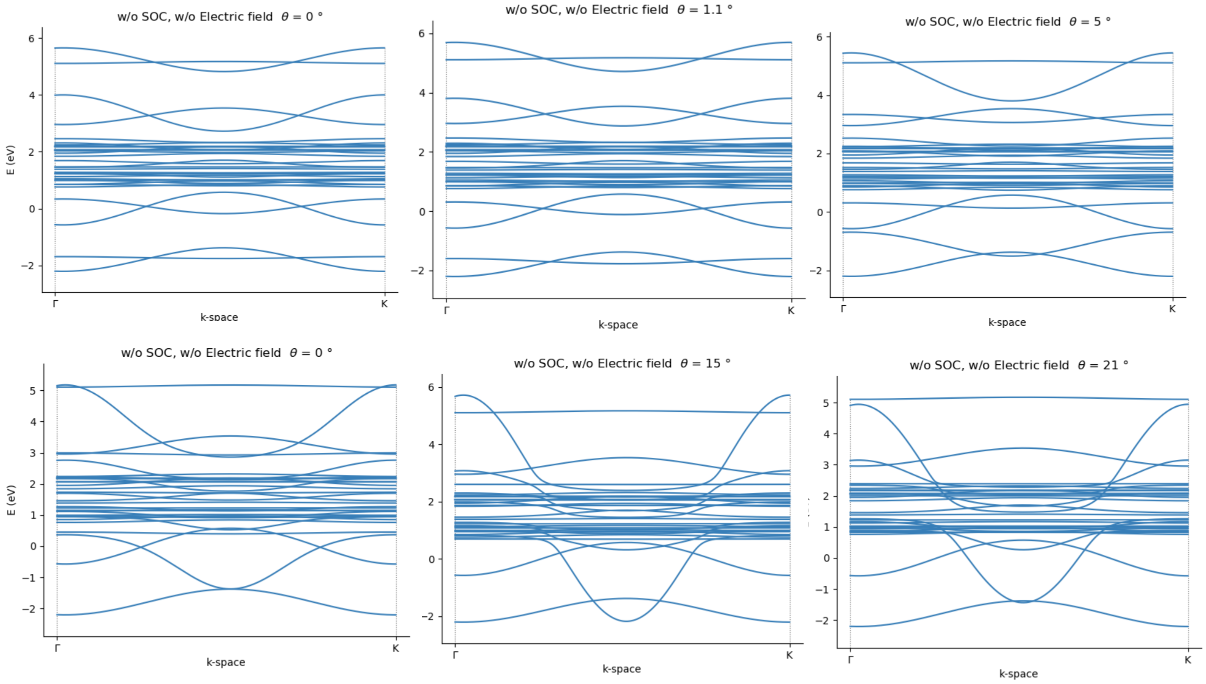

Fig. S4: Band structure of rectangular armchair hetero-nanoribbons WSe2/MoSe2 for twist-angles ($\theta = 0°, 1.1°, 5°, 9°, 15°,$ and $21°$) without spin-orbit coupling.

AC WSe_2/$MoSe_2$

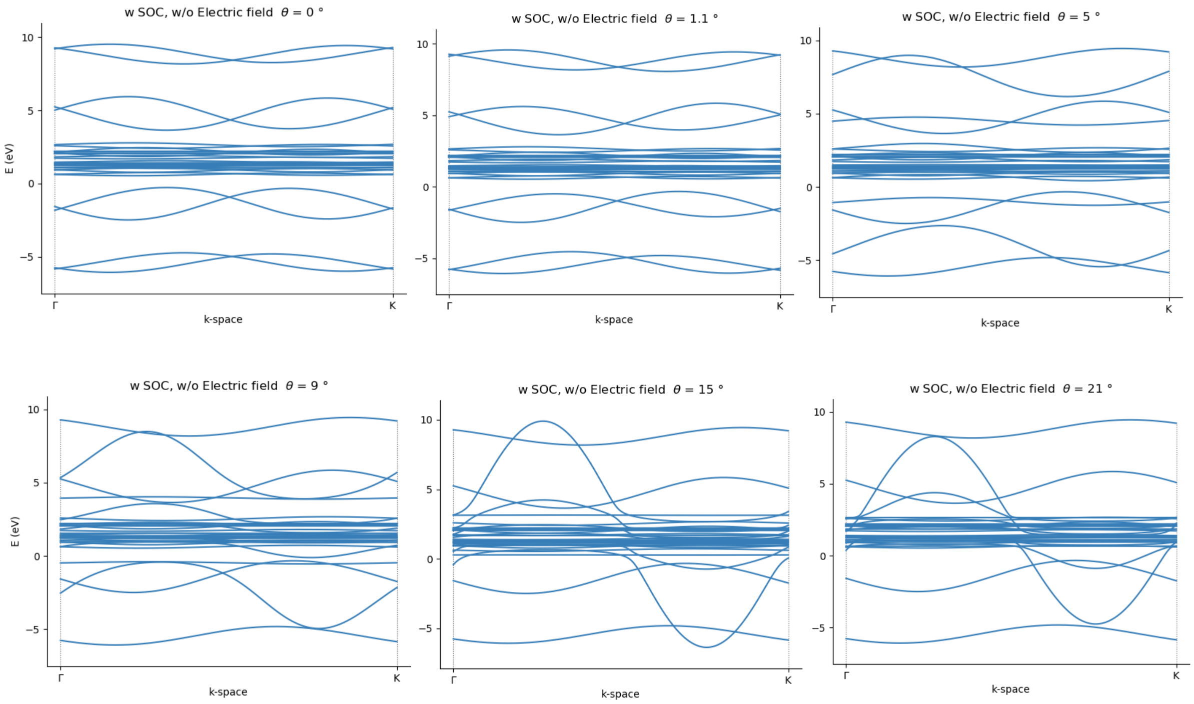

Fig. S 5: Band structure of rectangular armchair hetero-nanoribbons WSe2/MoSe2 for twist-angles ($\theta = 0°, 1.1°, 5°, 9°, 15°$, and $21°$) with spin-orbit coupling.

AC $WSe_2/MoSe_2$

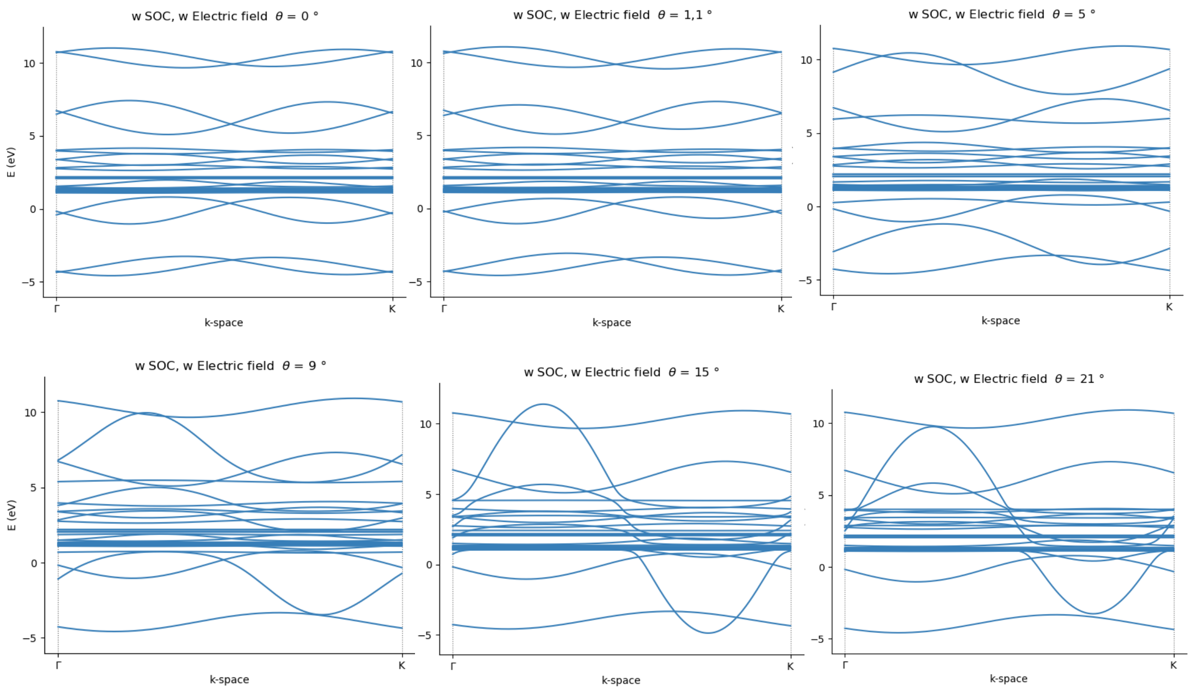

Fig. S6: Band structure of rectangular armchair hetero-nanoribbons WSe2/MoSe2 for twist-angles ($\theta = 0°, 1.1°, 5°, 9°, 15°,$ and $21°$) with spin-orbit coupling and with electric field (1 eV/nm).

ZZ WS_2/MoS_2

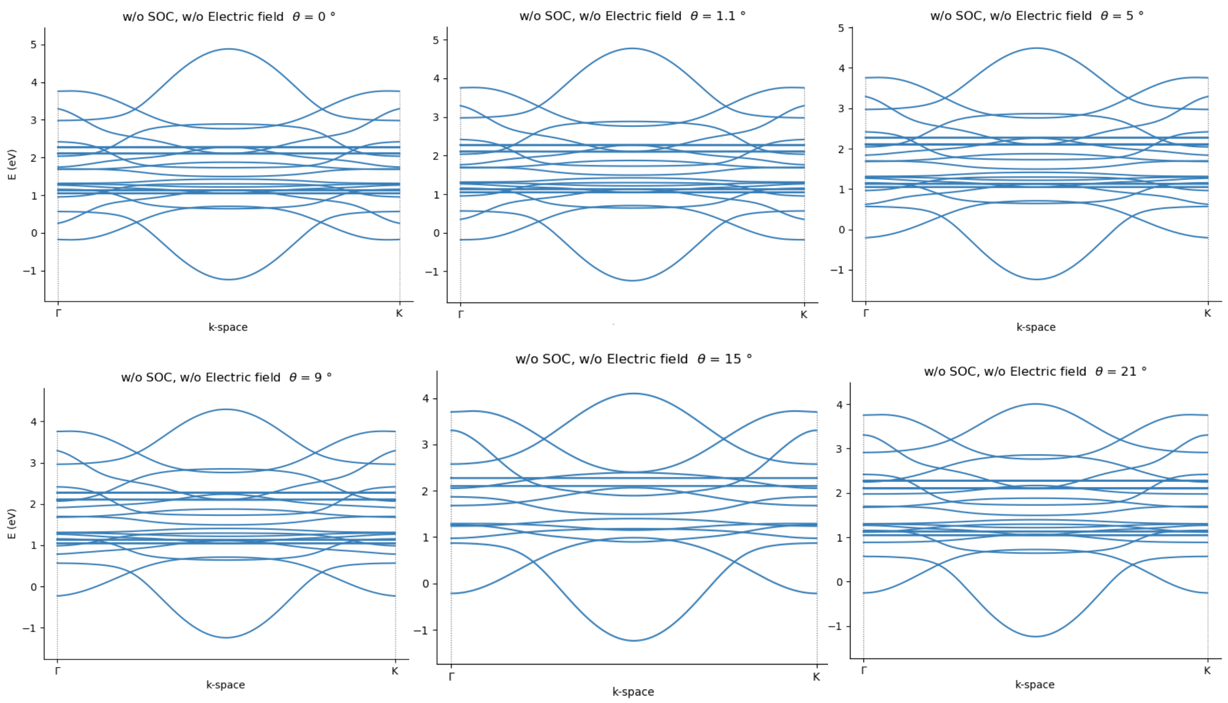

Fig. S7: Band structure of rectangular zigzag hetero-nanoribbons WS2/MoS2 for twist-angles ($\theta = 0°, 1.1°, 5°, 9°, 15°$, and $21°$) without spin-orbit coupling.

ZZ WS_2/MoS_2

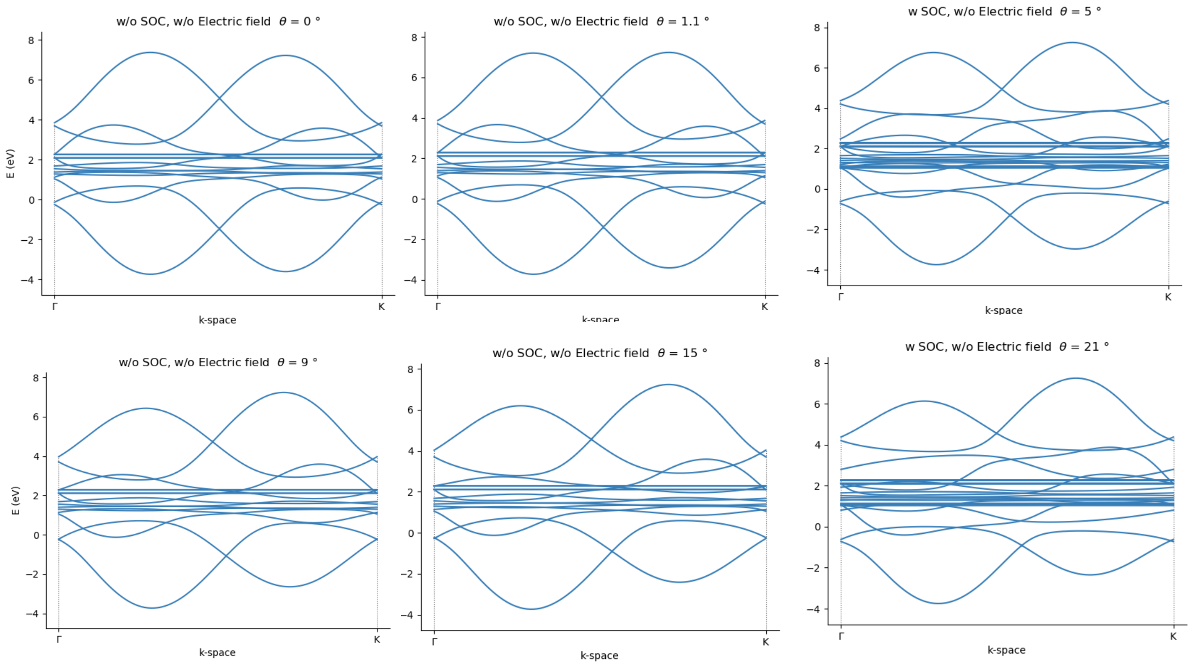

Fig. S8: Band structure of rectangular zigzag hetero-nanoribbons WS2/MoS2 for twist-angles ($\theta = 0°, 1.1°, 5°, 9°, 15°$, and $21°$) with spin-orbit coupling.

ZZ WS_2/MoS_2

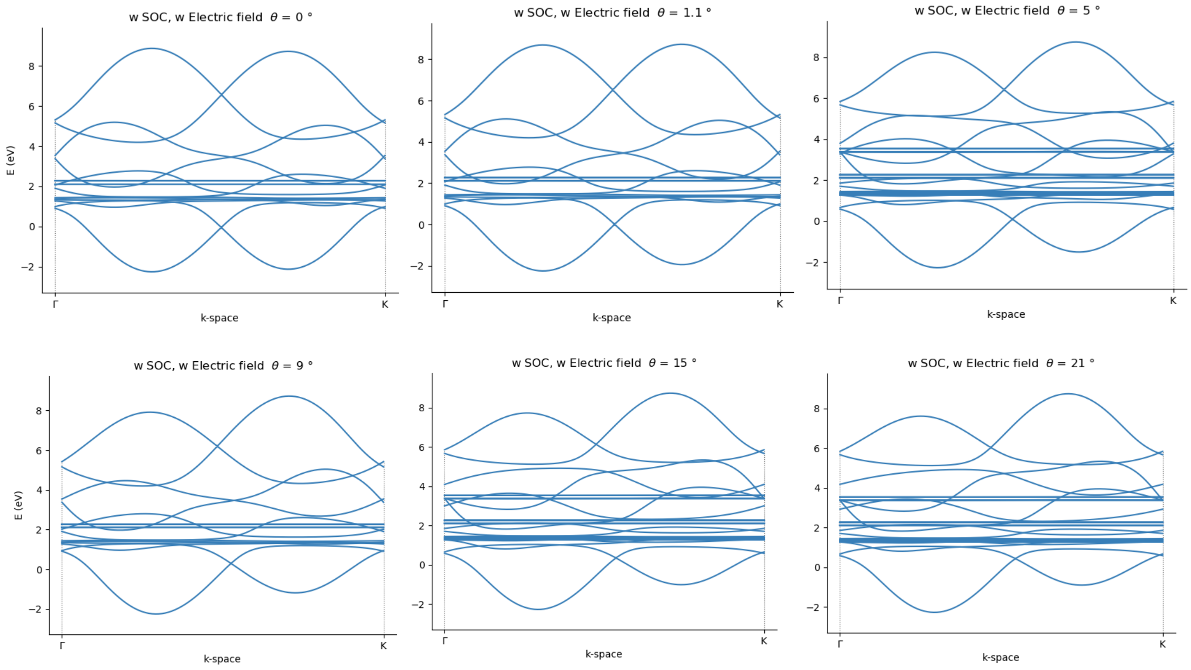

Fig. S9: Band structure of rectangular zigzag hetero-nanoribbons WS2/MoS2 for twist-angles ($\theta = 0°, 1.1°, 5°, 9°, 15°$, and $21°$) with spin-orbit coupling and with electric field (1 eV/nm).

ZZ $WSe_2/MoSe_2$

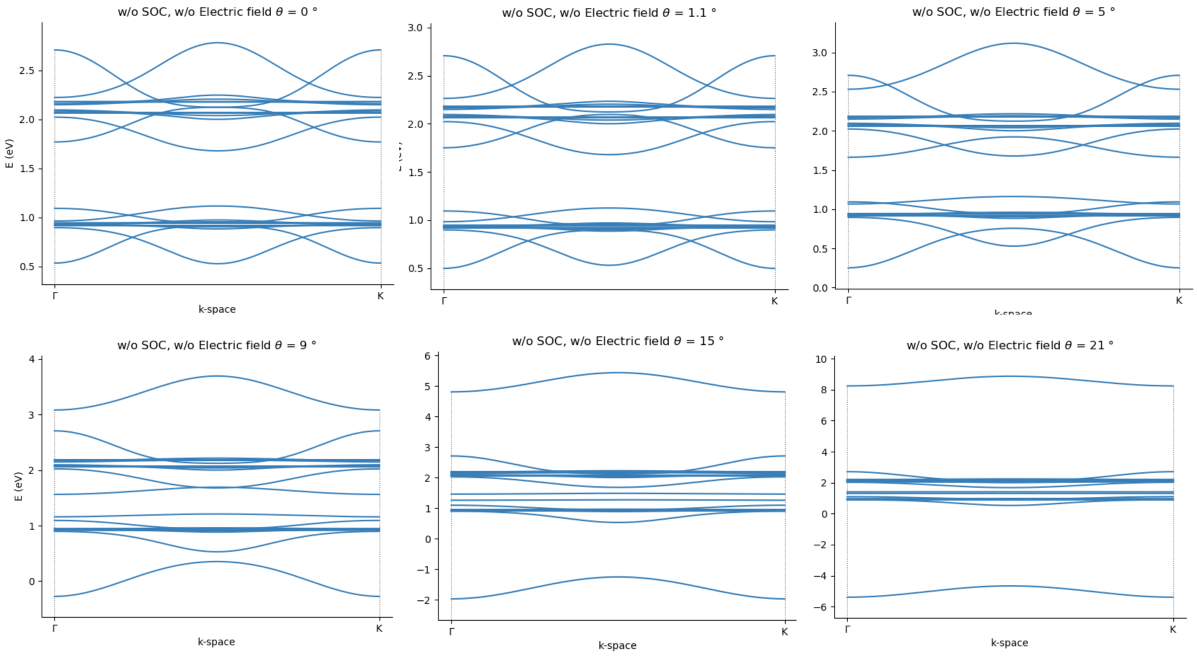

Fig. S 10: Band structure of rectangular zigzag hetero-nanoribbons WSe2/MoSe2 for twist-angles ($\theta = 0°, 1.1°, 5°, 9°, 15°$, and $21°$) without spin-orbit coupling.

ZZ $WSe_2/MoSe_2$

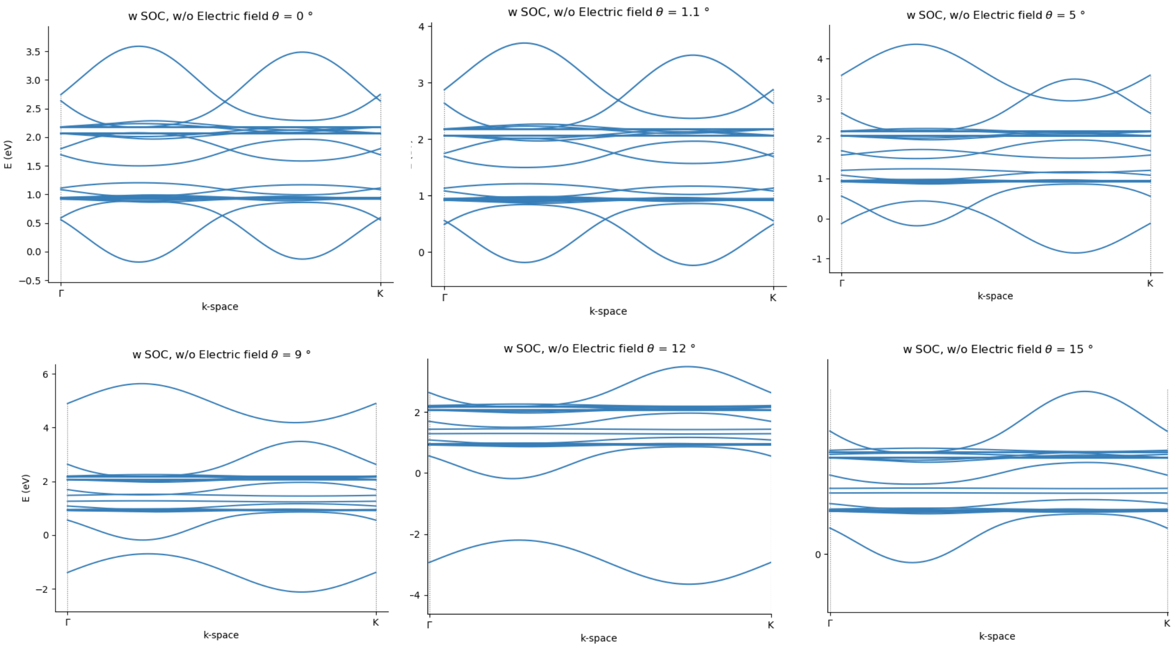

Fig. S 11: Band structure of rectangular zigzag hetero-nanoribbons WSe2/MoSe2 for twist-angles ($\theta = 0°, 1.1°, 5°, 9°, 15°$, and $21°$) with spin-orbit coupling.

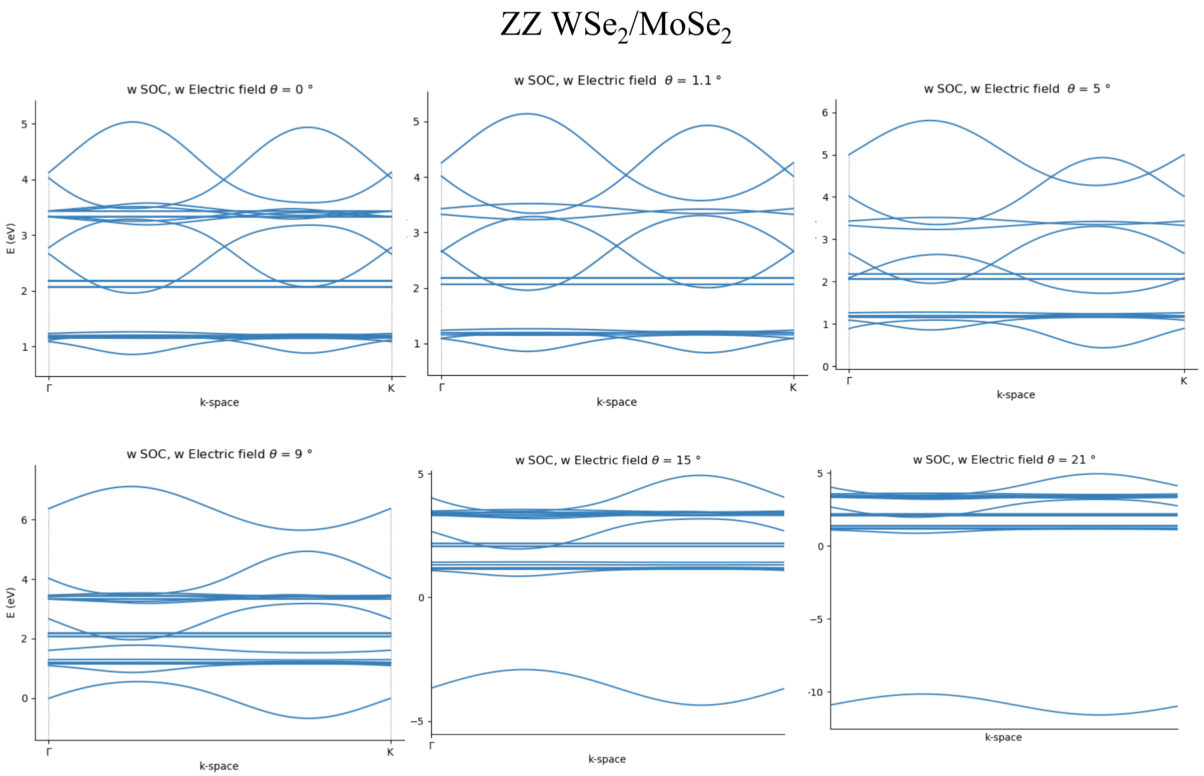

Fig. S 12: Band structure of rectangular zigzag hetero-nanoribbons WSe2/MoSe2 for twist-angles ($\theta = 0°, 1.1°, 5°, 9°, 15°$, and $21°$) with spin-orbit coupling and with electric field (1 eV/nm).

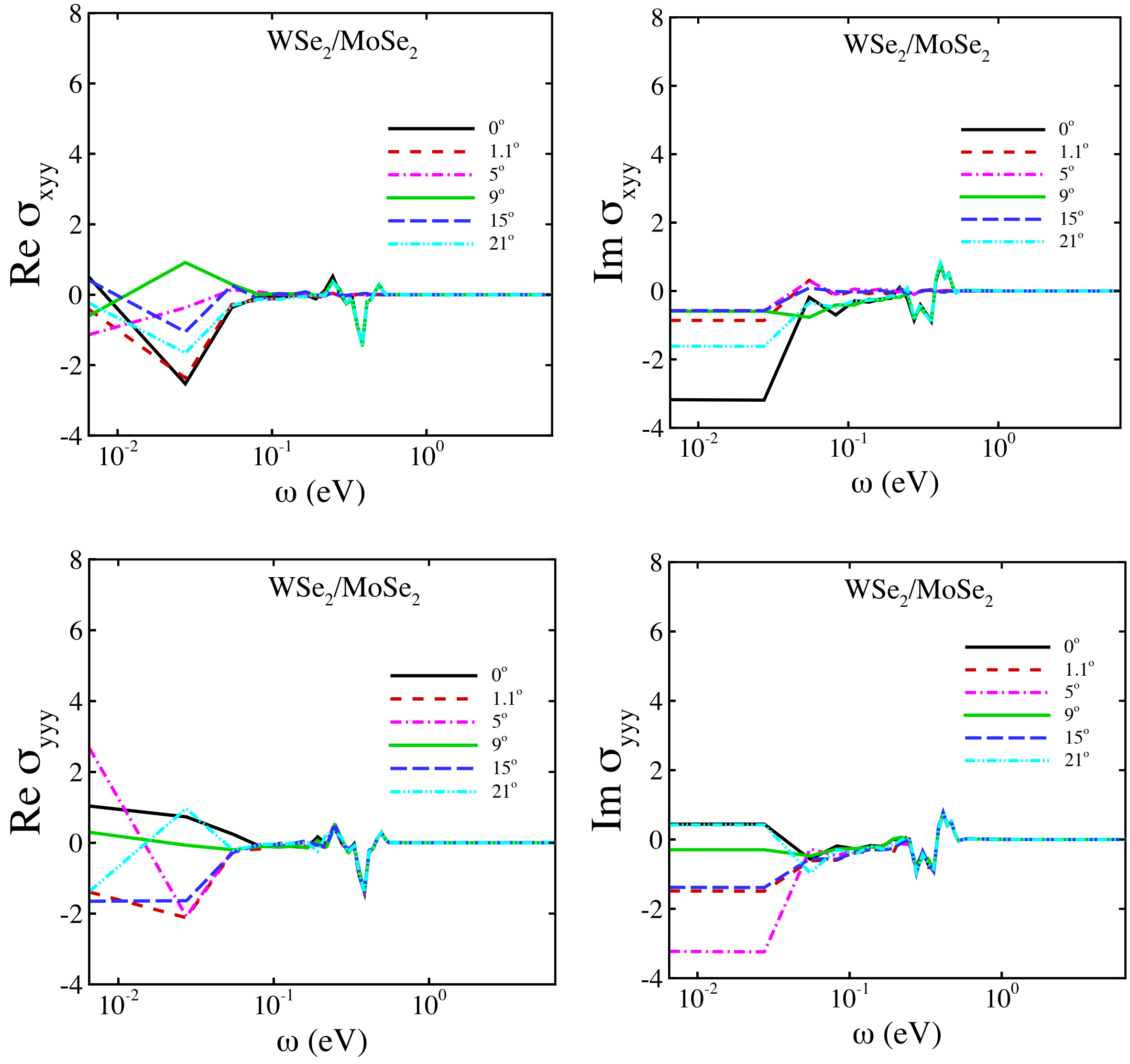

Fig. S13: Nonlinear quantum Hall effect of zigzag hetero-nanoribbons WSe2/MoSe2 for rotation angles of $\theta = 0°, 1.1°, 5°, 9°, 15°$, and $21°$. The top panels display the real and imaginary part of longitudinal conductivity σ_{xyy} and the bottom panels are for Hall conductivity σ_{yyy}. The gray line shows the terahertz region.

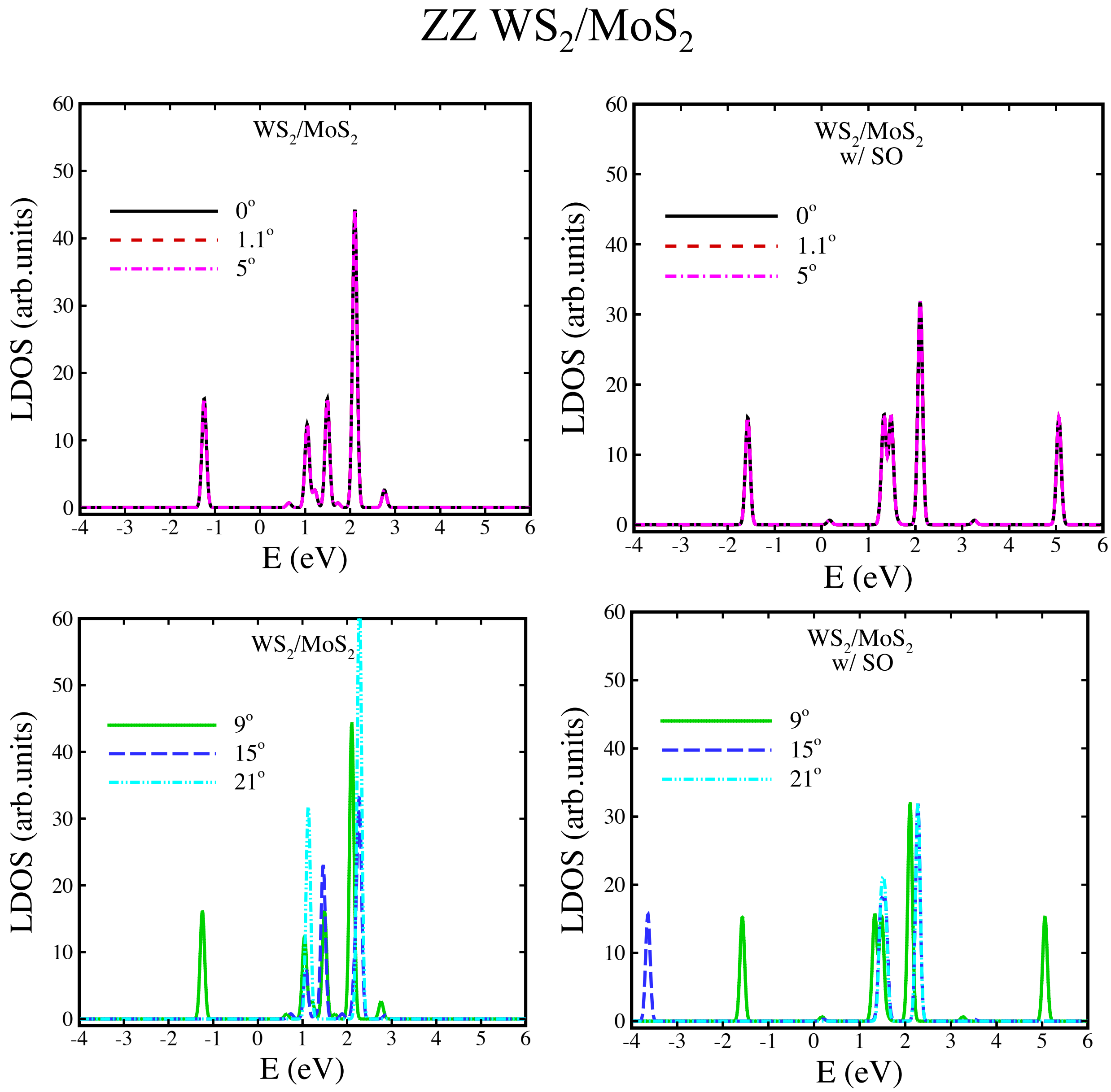

Fig. S14: Local density of states of zigzag hetero-nanoribbons WSe2/MoSe2 at twisted angles $\theta = 0°, 1.1°, 5°, 9°, 15°$.

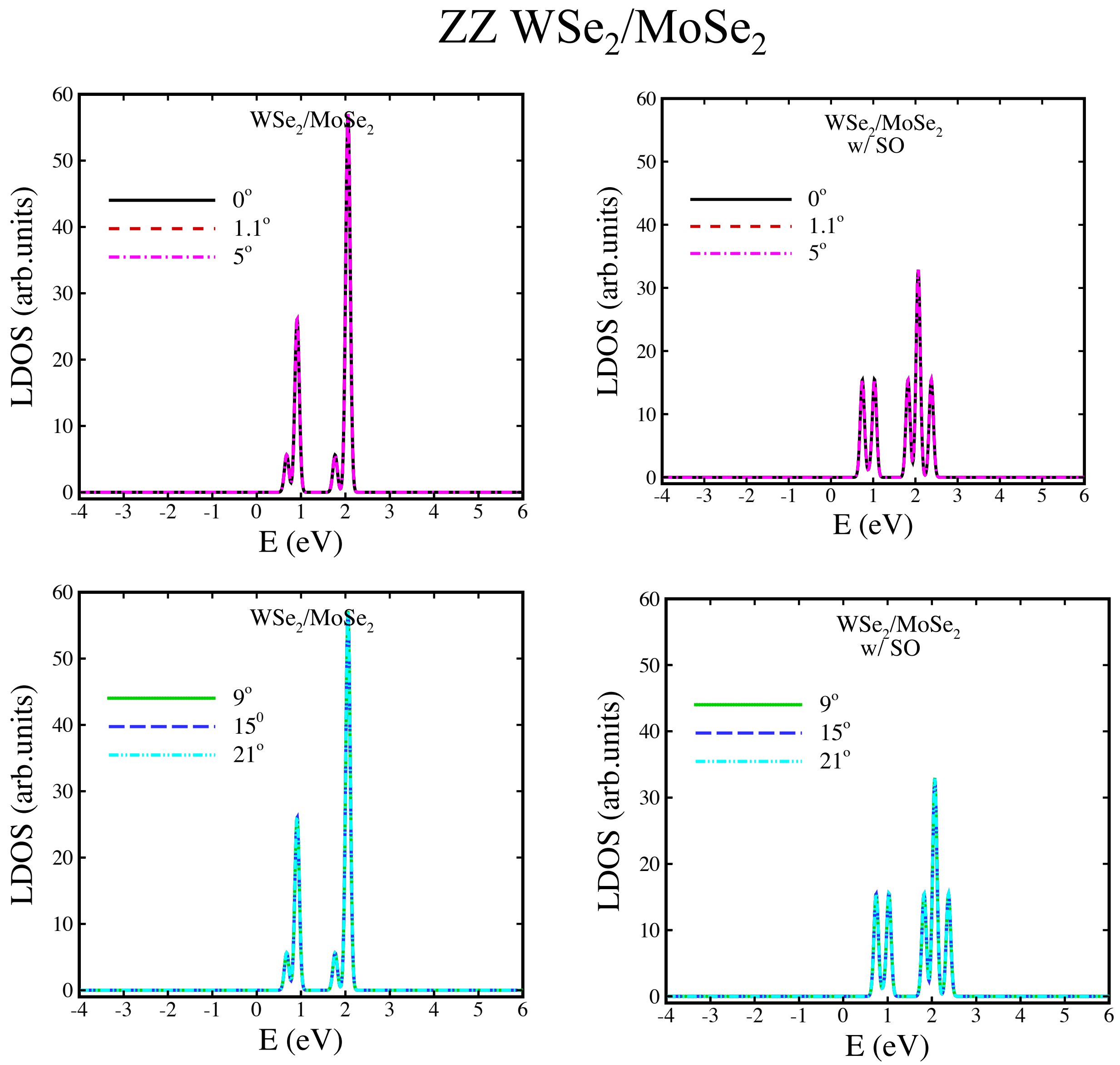

Fig. S 15: Local density of states of zigzag hetero-nanoribbons WS2/MoS2 at twisted angles $\theta = 0°, 1.1°, 5°, 9°, 15°$.

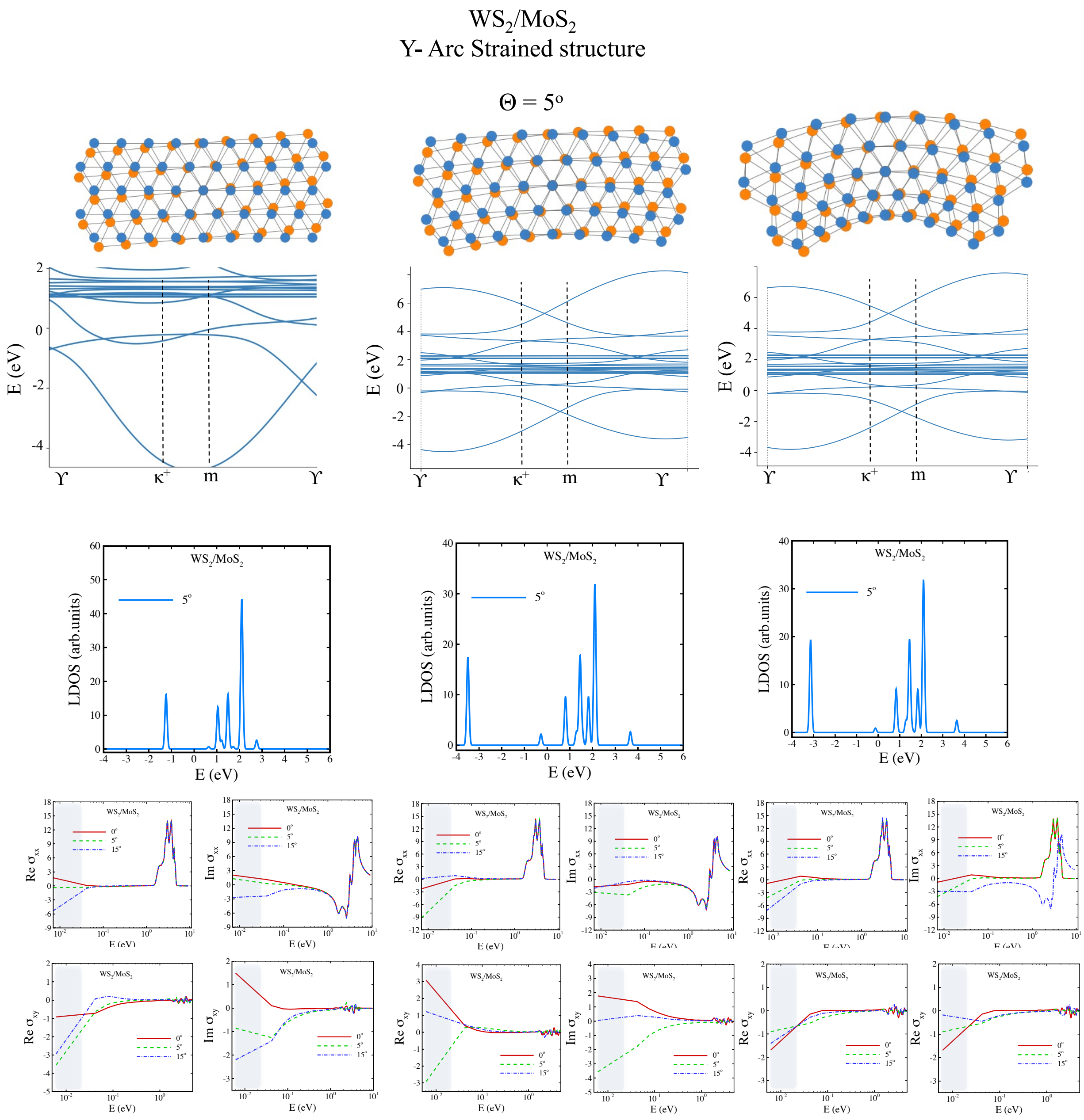
WS2/MoS2
Y- Arc Strained structure
Θ = 5°
E (eV)
ϒ
κ+
m
LDOS (arb.units)
5°
Re σxx
Im σxx
Re σxy
Im σxy
0°
15°

Fig. S 16

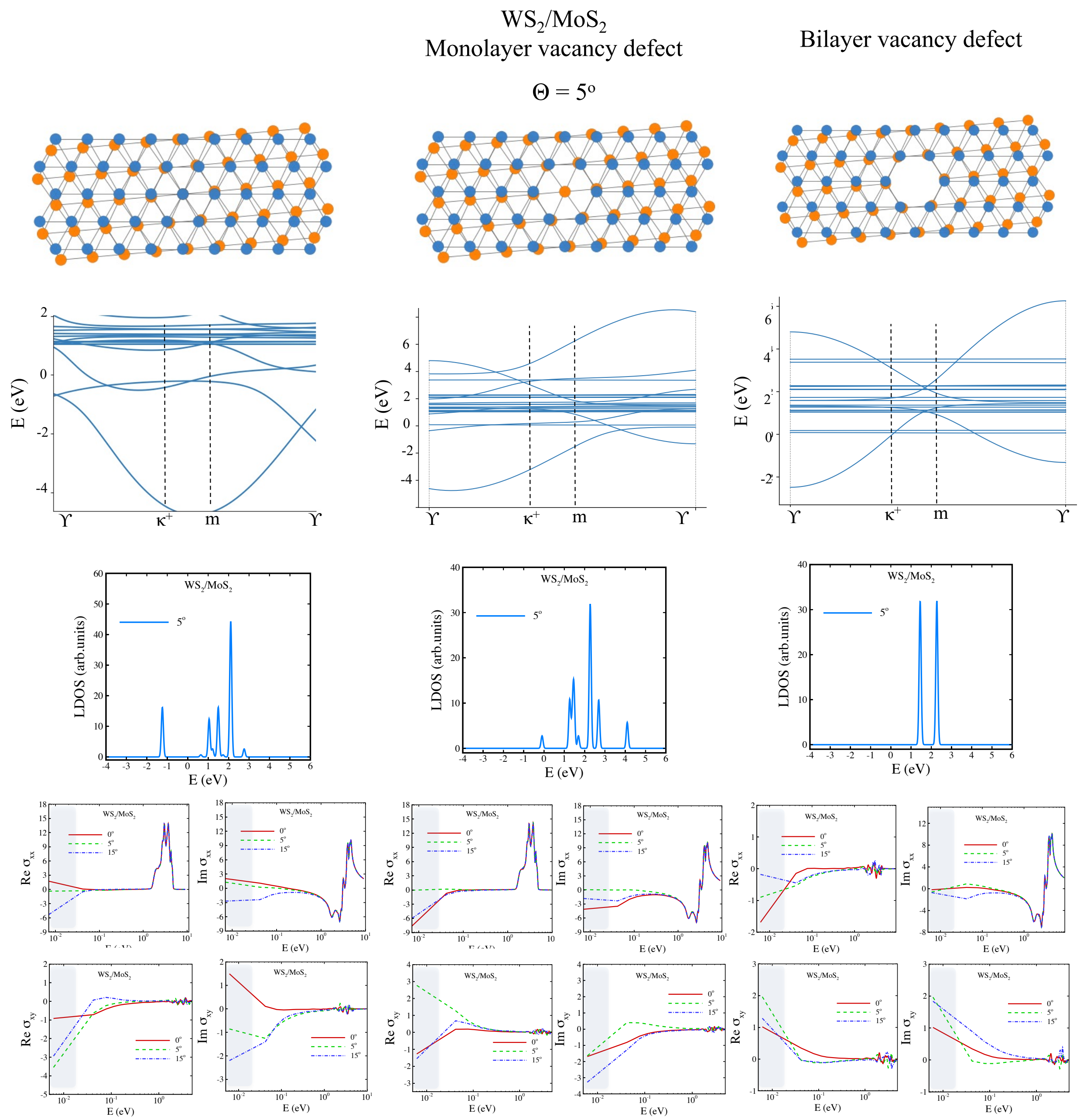
WS$_2$/MoS$_2$
Monolayer vacancy defect
Bilayer vacancy defect
Θ = 5°
E (eV)
Υ
κ+
m
LDOS (arb.units)
WS$_2$/MoS$_2$
5°
Re σxx
Im σxx
Re σxy
Im σxy
0°
5°
15°

Fig. S 17

.
